# Synthesis, Biological Evaluation, Molecular Docking and DFT Studies of Electronically Tuned Benzylidene-Based Schiff Bases

**DOI:** 10.3390/molecules31193519

**Published:** 2026-10-02

**Authors:** Kerem Buran, Elmas Rumeysa Yıldırım, Nazmiye Öner, Derya Doğanay, Esra Mertoğlu, Abdullahi İbrahim Uba, Gokhan Zengin

**Affiliations:** 1 Department of Pharmaceutical Chemistry, Hamidiye Faculty of Pharmacy, University of Health Sciences, Istanbul 34668, Turkey; elmasrumeysa@gmail.com; 2 Experimental Medicine Application & Research Center, Validebağ Research Park, University of Health Sciences, Istanbul 34662, Turkey; 3 Department of Pharmaceutical Chemistry, Institute of Graduate Studies in Health Sciences, Istanbul University, Istanbul 34116, Turkey; 4 Opticianry Program, Hamidiye Vocational School of Health Services, University of Health Sciences, Istanbul 34668, Turkey; nazmiye.oner@sbu.edu.tr; 5 Department of Pharmaceutical Microbiology, Hamidiye Faculty of Pharmacy, University of Health Sciences, Istanbul 34668, Turkey; ddoganay2904@gmail.com; 6 Department of Phytotherapy, Hamidiye Institute of Health Sciences, University of Health Sciences, Istanbul 34668, Turkey; esrmertoglu0@gmail.com; 7 Department of Biostatistics and Medical Informatics, Faculty of Medicine, İstinye University, Istanbul 34396, Turkey; abdullahi.iu2@gmail.com; 8 Department of Biology, Science Faculty, Selcuk University, Konya 42130, Turkey; gokhanzengin@selcuk.edu.tr

**Keywords:** antimicrobial activity, antioxidant activity, density functional theory, molecular docking, schiff base derivatives, α-Glucosidase inhibitors

## Abstract

Inspired by the medicinally relevant stilbene scaffold, a series of six electronically tuned benzylidene-based Schiff base derivatives were designed, synthesized, and characterized by FT-IR, ^1^H NMR, ^13^C NMR, and ESI-MS analyses. The biological properties of the synthesized compounds were investigated through antioxidant, enzyme inhibitory, and antimicrobial assays, while molecular docking and density functional theory (DFT) calculations were performed to rationalize the experimental findings and structure-activity relationships. Compounds **3** and **6** exhibited the strongest antioxidant activities, particularly in ABTS, CUPRAC, FRAP, phosphomolybdenum, and metal-chelating assays. Compounds **1**–**3** and **5** demonstrated measurable α-glucosidase inhibitory activity, although they were less potent than acarbose, whereas compounds **1** and **2** showed the highest tyrosinase inhibition within the synthesized series. Antimicrobial evaluation revealed moderate activity across the series, with compound **3** displaying relatively higher antimicrobial activity within the synthesized series. Docking studies demonstrated favorable interactions within enzyme active sites, while DFT calculations provided insights into electronic properties and molecular reactivity. As a result, the investigated substitution patterns were associated with differences in biological activity within the synthesized series, with compound **3** showing relatively favorable activity in selected assays and warranting further investigation.

## 1. Introduction

Schiff bases constitute one of the most important classes of imine-containing compounds in medicinal chemistry owing to their straightforward synthesis, structural diversity, and remarkable biological versatility. The azomethine (C=N) functionality provides an electronically tunable framework capable of interacting with diverse biological targets, making Schiff bases attractive scaffolds for drug discovery. Accordingly, numerous Schiff base derivatives have been reported to exhibit antimicrobial, antioxidant, anti-inflammatory, anticancer, antiviral, and enzyme inhibitory activities, establishing this class as a privileged pharmacophore in medicinal chemistry [1,2,3]. Among aromatic Schiff bases, N-benzylideneaniline derivatives represent a particularly attractive scaffold because they combine synthetic simplicity with a rigid diaryl-conjugated architecture. Structurally, benzylideneaniline can be regarded as an azastilbene analogue, in which the central carbon-carbon double bond of stilbene is replaced by an azomethine (C=N) linkage while preserving the overall diaryl geometry. This subtle bioisosteric modification retains molecular planarity and conjugation while introducing additional electronic tunability and hydrogen-bonding capability through the imine nitrogen, features that are highly desirable in medicinal chemistry. The selection of the benzylideneaniline scaffold in the present study was inspired by the well-established biological significance of stilbene-based natural products, particularly resveratrol and piceatannol. These compounds have attracted enormous interest because of their broad spectrum of biological activities, including antioxidant, antimicrobial, anti-inflammatory, anticancer, enzyme inhibitory, and tyrosinase inhibitory properties, making the stilbene framework one of the privileged structural motifs in medicinal chemistry [4,5,6]. Their diaryl-conjugated architecture has therefore served as a valuable template for the development of numerous bioactive small molecules. Indeed, the medicinal relevance of benzylideneaniline derivatives has already been demonstrated in several studies. Bae et al. successfully designed (*E*)-N-substituted benzylideneaniline derivatives as tyrosinase inhibitors by adopting a rational scaffold simplification strategy inspired by a biologically active aromatic template, demonstrating that even structurally simple benzylideneaniline derivatives can provide potent biological activity [7]. Likewise, benzylideneaniline derivatives have been reported to possess antiproliferative activity against human cancer cell lines, while N-benzylideneaniline itself has also been identified as an effective inhibitor of lignostilbene-α,β-dioxygenase, further supporting the capability of this scaffold to interact with biologically relevant enzyme systems [8,9].

**Figure 1:**
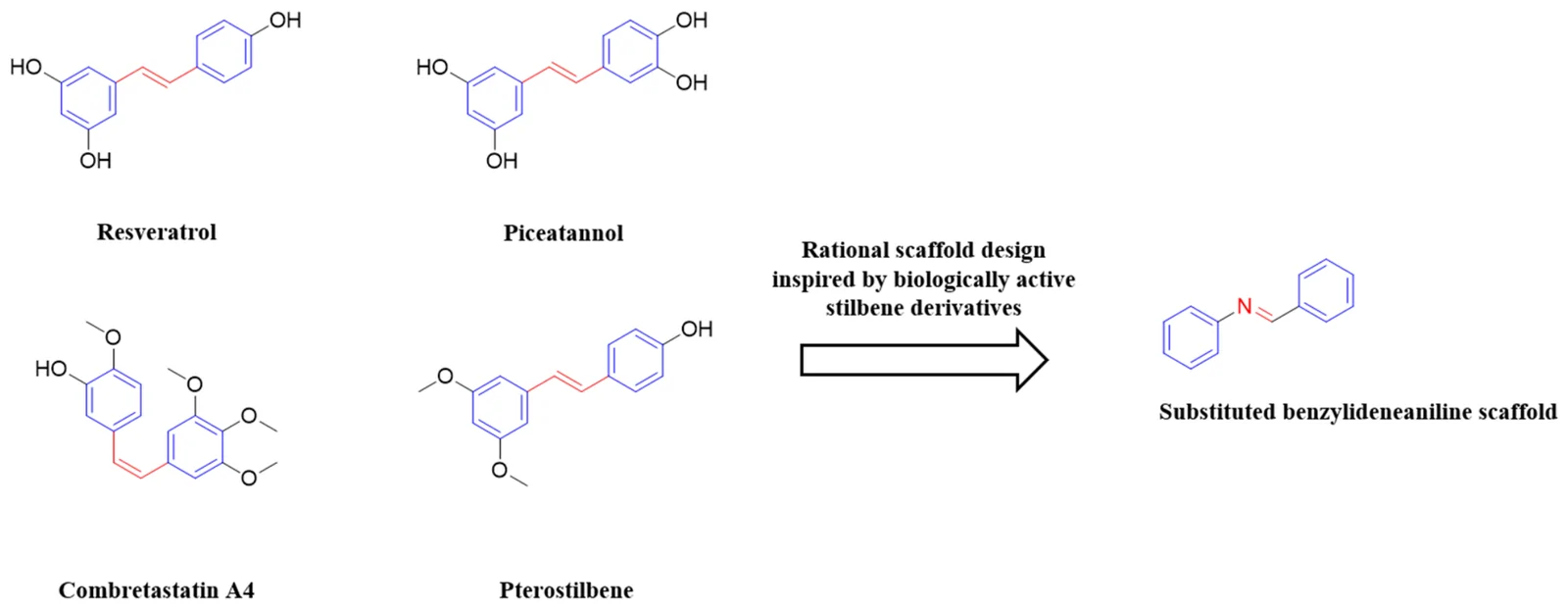
Design rationale for benzylideneaniline derivatives inspired by biologically active stilbenes.

Inspired by these previous medicinal chemistry studies, the rational design strategy adopted in the present study is illustrated in Figure 1. Accordingly, the benzylideneaniline framework was selected not merely as a classical Schiff base, but as a synthetically accessible azastilbene-inspired scaffold capable of retaining the favorable diaryl-conjugated architecture of stilbene while allowing straightforward structural diversification through simple substitution (Figure 1).

Unlike many previous studies focusing on a single biological target, the present work was designed to comprehensively investigate the biological potential of a representative series of *p*-substituted benzylideneaniline derivatives through integrated antioxidant, antimicrobial, and enzyme inhibitory evaluations supported by molecular docking and density functional theory (DFT) calculations. By combining experimental and computational approaches, this study aims to provide a broader medicinal chemistry perspective on this classical yet still underexplored aromatic imine scaffold.

## 2. Results and Discussions

### 2.1. Chemistry

**Scheme 1:**
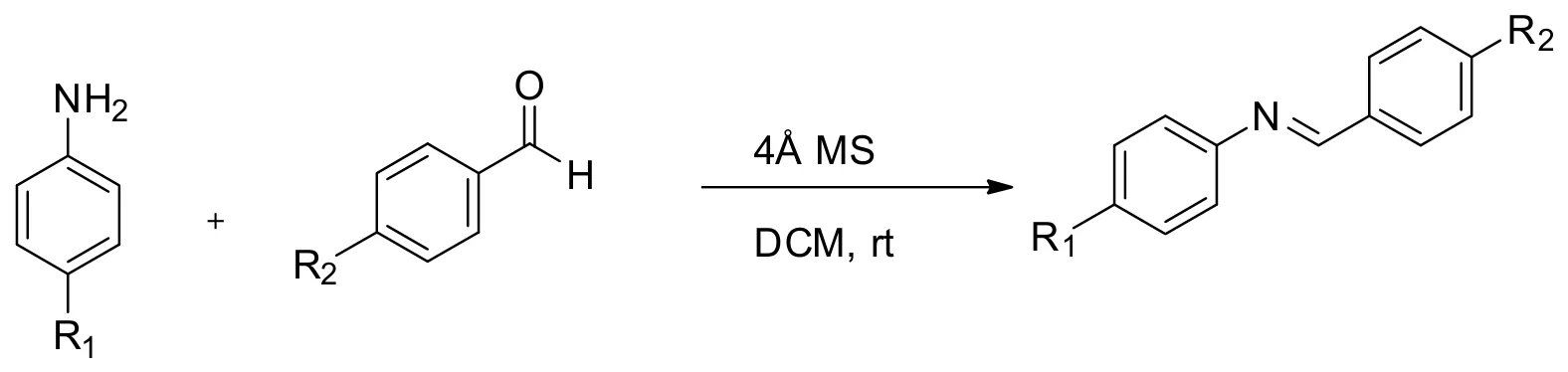
Synthetic pathway of compounds **1**–**6**.

The target Schiff base derivatives (**1**–**6**) were synthesized according to the reference [10]. Briefly, the corresponding substituted anilines and aromatic aldehydes reacting under mild conditions give condensation reactions as depicted Scheme 1. The reactions proceeded smoothly and afforded the desired products in good yields after simple work-up and purification procedures.

The imine framework was synthesized via nucleophilic addition of the amino group to the carbonyl carbon of the aldehyde with dehydrogenation to generate the characteristic azomethine scaffold (-CH=N-). The synthetic methodology proved efficient for both electron-donating and electron-withdrawing substituted substrates, yielding structurally diverse N-benzylideneaniline derivatives. Compounds **1**–**3** were composed of unsubstituted aniline moieties combined with phenyl, methoxyphenyl and dimethylaminophenyl aldehyde fragments; on the other hand, compounds **4**–**6** incorporate a p-chloroaniline scaffold (Table 1). The introduction of methoxy, dimethylamino, and chloro substituents was intended to modulate the electronic distribution of the azomethine system and provide valuable information regarding the influence of substituent effects on biological activity and molecular recognition. The synthesized compounds were characterized by FT-IR, ^1^H NMR, ^13^C NMR and ESI-MS.

### 2.2. Spectroscopic Characterization of Compounds ***1***–***6***

**Figure 2:**
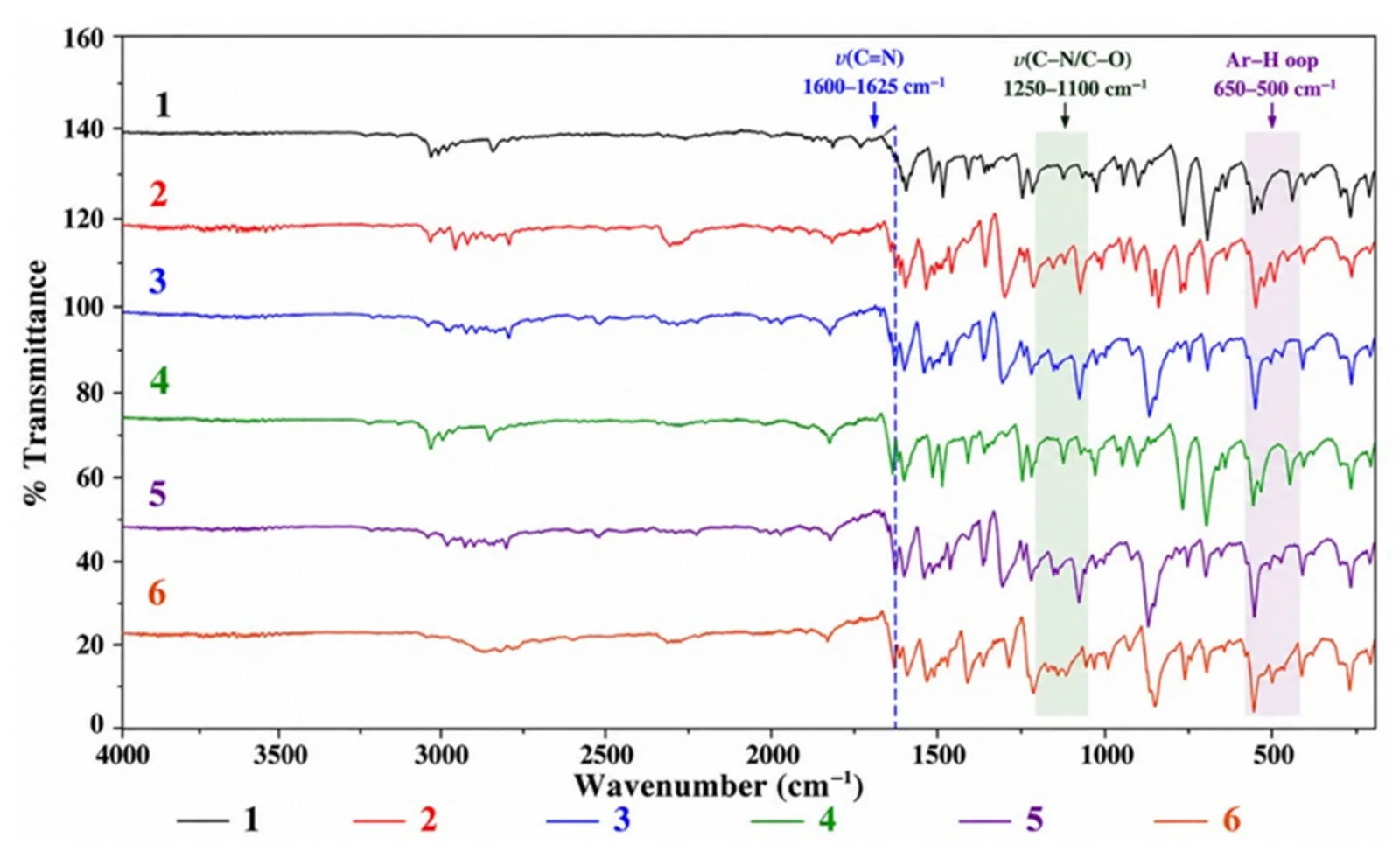
FT-IR spectra of the compounds **1**–**6.**

Molecular structures of the all-synthesized compounds (**1**–**6**) characterized and confirmed by FT-IR, ^1^H NMR, ^13^C NMR and ESI-MS. The FT-IR spectra of compounds (**1**–**6**) confirmed the successful formation of the target Schiff bases (Figure 2). The characteristic azomethine stretching vibrations were observed at 1601–1625 cm^−1^. Specifically, compounds **1** and **4** exhibited ν(C=N) bands at approximately 1625 cm^−1^, whereas compounds **2**, **3**, **5**, and **6** displayed this absorption between 1601 and 1602 cm^−1^, indicating the formation of the imine linkage. In addition, compounds **1** and **4** displayed a characteristic azomethine C-H stretching vibration at approximately 2892 cm^−1^. The vibrations of the aromatic C-H were observed in the 3000–3061 cm^−1^. Additional bands which were detected between 1242 and 1350 cm^−1^ were referred to C-N and/or C-O stretching vibrations. Moreover, characteristic aromatic C-H out-of-plane (oop) bending vibrations were assigned in the 690–838 cm^−1^. The overall FT-IR data were in good agreement with the proposed molecular structures and provided strong spectroscopic evidence for the successful synthesis of the Schiff base derivatives. Furthermore, the absence of the amino (-NH_2_) and aldehyde carbonyl stretching bands of the starting materials together with the appearance of new absorption bands corresponding to the azomethine functionality confirmed successful Schiff base formation. All compounds exhibited a characteristic ν(C=N) stretching vibration within the expected region for aromatic imines.

Aromatic C=C stretching vibrations were also observed, while compounds bearing methoxy substituents displayed additional ν(C-O-C) absorptions. The chloro-substituted derivatives exhibited characteristic bands associated with C-Cl vibrations. The overall similarity observed among the FT-IR spectra of compounds **1**–**6** is attributed to their common benzylideneaniline (Schiff base) framework, which gives rise to similar characteristic vibrational bands. Nevertheless, subtle spectral differences associated with the substituents, particularly the methoxy, dimethylamino, and chloro groups, were observed in the corresponding characteristic regions.

The next characterization technique was NMR spectroscopy. The ^1^H NMR spectra of compounds **1**–**6** provided further evidence for the formation of the proposed Schiff base structures. In the ^1^H NMR spectra, all compounds had characteristic singlets corresponding to azomethine proton (-CH=N) in the range δ 8.38–8.61 ppm. The E-configuration was considered the most plausible based on the characteristic downfield chemical shift of the azomethine proton (δ 8.38–8.61 ppm) and the thermodynamic preference for the E-isomer under the employed reaction conditions. However, since NOE/ROESY or single-crystal X-ray analyses were not performed to directly confirm the C=N geometry, no definitive E/Z assignment was made, and the stereochemical designation was therefore omitted from the nomenclature and structural representations. The signals of the aromatic protons were found between δ 6.76 and 7.93 ppm which were suitable splitting patterns according to the substitution pattern of the phenyl rings. Compounds **2** and **5** had additional singlets at δ 3.82 and 3.81 ppm respectively, that referred to methoxy protons, whereas methyl protons of the compounds **3** and **6** were observed at δ 2.99–3.00 ppm. The ^13^C NMR spectra of compounds displayed characteristic imine carbon signals in the range of δ 150.6–152.7 ppm region. Signals of highly deshielded substituted aromatic carbons were assigned between δ 160.3 and 162.5 ppm, whereas the other carbons were observed in the expected region δ 111.5–136.4 ppm. Although the methyl proton of compounds **3** and **6** signals were clearly observed in the ^1^H NMR, the corresponding methyl carbon resonance could not be distinguished in the ^13^C NMR. It may be caused by signal overlap of DMSO or limited resolution in the aliphatic region. Although the dimethylamino methyl proton signals of compounds **3** and **6** were clearly observed in the ^1^H NMR spectra at δ 2.99–3.00 ppm, the corresponding ^13^C resonance could not be reliably distinguished, possibly due to signal overlap in the DMSO-d_6_/aliphatic region and the limited resolution of the recorded spectra. Therefore, no definitive assignment of this carbon resonance was made. As a result, the observed proton and carbon resonances were in full agreement with the proposed structures of compounds **1**–**6**. Lastly, synthesized compounds were analyzed with ESI-MS to confirm molecular structures. The experimentally observed molecular ion peaks were in excellent agreement with the calculated molecular weights, providing additional confirmation of the successful synthesis of the target compounds. Taken together, the combined FT-IR, ^1^H NMR, ^13^C NMR, and ESI-MS data were consistent with and supported the proposed structures of compounds **1–6**. In addition, the NMR spectra did not show prominent signals attributable to major impurities, supporting the satisfactory purity of the isolated compounds.

### 2.3. Biological Activity

#### 2.3.1. Antioxidant Activity

The compounds (**1**–**6**) were evaluated for antioxidant properties using complementary assays based on different antioxidant mechanisms. DPPH, ABTS radical scavenging, CUPRAC, FRAP reducing power, phosphomolybdenum total antioxidant capacity, and metal-chelating activity were applied (Table 2). Results revealed that the antioxidant capacity of synthesized Schiff bases strongly depend on the nature of the aromatic substituents.

For DPPH radical scavenging activity, none of the compounds exhibited remarkable results for tested concentrations. This means that compounds (**1**–**6**) have limited hydrogen atom donation ability which is generally required for DPPH activity. In contrast, notable activities were observed in the ABTS, CUPRAC, FRAP, phosphomolybdenum and metal-chelating assays. Among the compounds, **3** and **6** were the most promising antioxidants. Compound **3** exhibited the strongest ABTS radical scavenging activity (IC_50_ = 2.20 ± 0.06 mg/mL) and the highest CUPRAC reducing capacity (IC_50_ =1.32 ± 0.04 mg/mL), indicating an enhanced electron-donating ability (Table 2). The superior performance of compound **3** can be attributed to the presence of the strongly electron-donating dimethylamino substituent, which increases the electron density of the conjugated imine framework and facilitates electron transfer processes involved in radical neutralization. Likewise, compound **6** demonstrated pronounced antioxidant potential in several assays (Table 2). In the ABTS assay, compound **6** had the lowest IC_50_ value (IC_50_ = 2.35 ± 0.06 mg/mL) and notable activities were found for FRAP (IC_50_ = 1.67 ± 0.03 mg/mL), phosphomolybdenum (IC_50_ = 1.14 ± 0.14 mg/mL), and metal-chelating assays (IC_50_ = 0.72 ± 0.02 mg/mL). The enhanced activity of compound **6** may arise from the synergistic electronic effects of the p-dimethylamino and p-chloro substituents, which can modulate the electron distribution of the azomethine-containing π-system and this may result favoring both redox processes and metal ion coordination. In contrast, compounds **1**, **2** and **4** had relatively weak antioxidant capacity (Table 2). Compound **2**, which had methoxy substituent exhibited only moderate ABTS activity and was inactive remaining assays. Similarly, compound **4**, containing a p-chloro substituent, displayed limited radical scavenging and reducing capacities. This suggests that the electron-withdrawing character of chlorine alone is not enough to promote efficient antioxidant behavior. Lastly, compound **5** was among the least active compound, it demonstrated weak and negligible activities in most assays despite the presence of methoxy and chloro substituents. To sum up, results indicate that strong electron-donating group, particularly the dimethylamino group, significantly improves the antioxidant capacity of synthesized Schiff bases. Taken together, the observed assay-dependent activity pattern suggests differences in the radical-scavenging and reducing capacities of the synthesized compounds; however, these indirect chemical assays do not permit definitive assignment of a specific antioxidant mechanism. These results suggest preliminary substituent-dependent trends in antioxidant activity within the investigated series.

#### 2.3.2. Enzyme Inhibitory Activity

The enzyme inhibition potential of compounds **1**–**6** were evaluated for tyrosinase, α-amylase and α-glucosidase enzymes and results summarized in Table 3. In enzyme inhibition assays results were given as IC_50_ (mM), kojic acid and acarbose were used as reference molecules. In the tyrosinase inhibition assay, compounds **1** and **2** showed the highest inhibitory activities among the synthesized derivatives, with IC_50_ values of 8.44 ± 0.06 and 7.76 ± 0.14 mM, respectively. Compound **3** showed lower activity (IC_50_ = 11.19 ± 0.40 mM), while compound **5** exhibited the weakest measurable tyrosinase inhibition (IC_50_ = 13.72 ± 2.40 mM). Compounds **4** and **6** were inactive under the tested conditions. All active synthesized compounds were substantially less potent than the reference inhibitor kojic acid (IC_50_ = 0.49 ± 0.01 mM). Within the synthesized series, the unsubstituted and methoxy-containing derivatives showed relatively higher tyrosinase inhibitory activity than the other investigated substitution patterns. However, considering the limited number of derivatives, these observations should be regarded as preliminary rather than as evidence of a definitive structure-activity relationship.

In the α-amylase inhibition assay, compound **4** exhibited the highest inhibitory activity among the synthesized derivatives, with an IC_50_ value of 5.89 ± 0.14 mM, followed by compounds **5** (6.23 ± 1.38 mM), 2 (6.25 ± 0.05 mM), and **1** (7.06 ± 0.22 mM). Compounds **3** and **6** were inactive under the tested conditions. All active synthesized compounds were considerably less potent than the reference inhibitor acarbose (IC_50_ = 0.94 ± 0.02 mM). Within the investigated series, the relatively similar activities of compounds **2**, **4**, and **5** suggest that different substitution patterns can be tolerated for α-amylase inhibition. In contrast, the dimethylamino-containing compounds **3** and **6** were inactive, suggesting that this substitution pattern may be unfavorable for α-amylase inhibitory activity within the present series.

In the α-glucosidase assay, compounds **1**–**5** exhibited measurable inhibitory activity, whereas compound **6** was inactive under the tested conditions. Among the synthesized derivatives, compound **5** showed the highest inhibitory activity with an IC_50_ value of 4.31 ± 0.04 mM, followed by compounds **3** (4.50 ± 0.04 mM), **2** (4.88 ± 0.05 mM), and **1** (5.57 ± 0.06 mM). Compound **4** exhibited considerably weaker activity, with an IC_50_ value of 12.61 ± 0.65 mM. In comparison, the reference inhibitor acarbose showed an IC_50_ value of 1.70 ± 0.11 mM, demonstrating that all active synthesized compounds were less potent than acarbose on a molar basis. Within the investigated series, compounds **1**–**3** and **5** showed relatively higher α-glucosidase inhibitory activity than compound **4**, whereas compound **6** was inactive. These findings indicate that the substitution pattern influences α-glucosidase inhibition; however, the limited number of derivatives does not allow a definitive structure–activity relationship to be established.

Considering all the findings, the enzyme inhibition results revealed that the biological responses of the investigated Schiff base derivatives varied depending on both the enzyme type and the substitution pattern. Compounds **1** and **2** showed relatively higher tyrosinase inhibitory activity within the synthesized series, while compounds **4**, **5**, and **2** exhibited the highest α-amylase inhibitory activities. For α-glucosidase, compound **5**, followed by compounds **3** and **2**, showed the highest inhibitory activity among the synthesized derivatives. However, comparison of the IC_50_ values on a molar basis demonstrated that the synthesized compounds were less potent than the corresponding reference inhibitors. Interestingly, compound **6**, despite showing comparatively high antioxidant activity within the series, was inactive against all three tested enzymes, indicating that the structural features associated with antioxidant activity do not necessarily result in enzyme inhibitory activity. As a result, these findings indicate substitution-dependent differences in enzyme inhibition within the investigated series and may provide a basis for further structural optimization of the Schiff base scaffold. However, the observed activity differences cannot be attributed exclusively to electronic effects, as steric, lipophilic, conformational, solvation, and other physicochemical properties may also contribute to the biological responses. Therefore, the substituent-dependent observations in the present limited series should be regarded as preliminary trends rather than a quantitative SAR.

The present study evaluated enzyme inhibition using IC_50_ values and did not include detailed enzyme kinetic experiments; therefore, a specific mode of inhibition (competitive, non-competitive, or mixed) and Ki values cannot be assigned from the current data. In addition, specific aggregation or precipitation-control experiments were not performed; therefore, nonspecific effects related to compound aggregation or limited solubility at the relatively high test concentrations cannot be completely excluded. Further kinetic and aggregation-control studies will be required to establish the inhibition mechanism of the active compounds.

#### 2.3.3. Antimicrobial Activity

The antibacterial and antifungal activities of compounds **1–6** were evaluated against a panel of Gram-positive bacteria, Gram-negative bacteria and yeast strains. The obtained minimum inhibitory concentration (MIC) values are presented in Table 4. Generally, the synthesized compounds exhibited moderate antimicrobial activity, with MIC values ranging from 250 to 1000 μg/mL against Gram-positive bacteria, 250 to 500 μg/mL against Gram-negative bacteria, and 500 to 1000 μg/mL against yeast strains. Most of the tested compounds (**1**, **2**, **4**, **5** and **6**) displayed MIC values within the range of 500–1000 μg/mL against the majority of the investigated microorganisms, whereas compound **3** demonstrated comparatively stronger antimicrobial activity, exhibiting MIC values between 250 and 1000 μg/mL.

The lowest MIC value observed in this study was 250 μg/mL, recorded for compound 3 against *E. coli* ATCC 25922 and *E. faecalis* ATCC 29212. Thus, compound **3** exhibited relatively higher antimicrobial activity within the synthesized series. However, the MIC values of the synthesized compounds remained substantially higher than those of the reference antimicrobial agents, indicating only modest antimicrobial activity. Therefore, these findings should be considered preliminary and do not establish compound **3** as a potent antimicrobial lead. 

In general, the tested compounds showed modest antimicrobial activity. Among the Gram-positive bacteria, *S. epidermidis* ATCC 12228 exhibited MIC values of 1000 μg/mL for all tested compounds, whereas *E. faecalis* ATCC 29212 showed a lower MIC value of 250 μg/mL for compound **3**. Among the Gram-negative bacteria, *E. coli* ATCC 25922 exhibited an MIC value of 250 μg/mL for compound **3**, while A. baumannii ATCC 19606 and *P. aeruginosa* ATCC 27853 generally displayed MIC values of 500 μg/mL. The reference antimicrobial agents exhibited substantially lower MIC values than the synthesized compounds. Thus, compound **3** showed relatively higher antimicrobial activity within the synthesized series against selected bacterial strains; however, its activity remained modest compared with the reference agents. Only MIC values were determined in the present study; MBC and MFC were not evaluated. Therefore, the present findings reflect growth-inhibitory activity under the tested conditions and do not permit conclusions regarding bactericidal or fungicidal effects.

### 2.4. Molecular Docking

#### 2.4.1. Predicting Binding Energy

Molecular docking studies were performed against tyrosinase (PDB ID: 6QXD), α-amylase (PDB ID: 1B2Y), and α-glucosidase (PDB ID: 7KBJ) to investigate the predicted binding modes and ligand–protein interactions of compounds **1**–**6**. The docking scores of compounds **1**–**6** against tyrosinase, α-amylase, and α-glucosidase are expressed in kcal/mol, representing estimated binding energies that reflect the binding strength of enzyme-ligand interactions [11,12]. Lower binding energies in docking-based studies denote stronger ligand-protein interactions and higher thermodynamic stability of the complex and are used as an initial predictor of the inhibitory potential [13]. The binding energies of the compounds were in the range of −4.02 to −6.50 kcal/mol, suggesting moderate affinity to all targets compared with highly potent inhibitors normally reported in the literature (Table 5) [14,15].

Compounds **4** and **2** showed the best binding energy values, −6.50 kcal/mol and −5.95 kcal/mol, respectively, for tyrosinase, suggesting that the compounds possess structural features that enable them to fit more efficiently in the enzyme’s active site. The catalytic pocket of these interactions is stabilized by a combination of hydrogen bonding, π-π stacking, and hydrophobic contacts, which are critical determinants of tyrosinase inhibition. In comparison, binding of compound **5** and compound **6** was less favorable (∼−4.0 kcal/mol), suggesting suboptimal complementarity with the enzyme and possibly lower inhibitory activity. The docking profiles against α-amylase revealed a relatively narrow range of binding energies (−5.06 to −5.73 kcal/mol), suggesting similar interaction patterns across the compound series. Compound **2** showed the highest affinity (−5.73 kcal/mol), followed by compound **1** (−5.68 kcal/mol), and compound **4** was the weakest binder in this group. However, the observed energies are not as good as the ones reported for standard inhibitors like acarbose, which normally have binding energies around −8.5 kcal/mol [16]. This means that although the compounds **1**–**6** compounds are capable of binding to α-amylase, their inhibitory potency might be limited, and that structural optimization is required to enhance enzyme binding and specificity. A similar pattern was found for α-glucosidase with moderate binding affinities of most compounds (−5.60 to −5.76 kcal/mol). The binding energies of −5.76 and −5.75 kcal/mol were obtained for compound **1** and compound **2**, respectively, indicating that they are the best candidates and have relatively strong interactions with the enzyme. On the contrary, the binding energy of compound **6** was significantly lower (−4.41 kcal/mol), indicating a weaker interaction with the catalytic site. The clustering of the docking scores within a relatively narrow range for α-amylase and α-glucosidase can be attributed to the common benzylideneaniline scaffold shared by compounds **1**–**6**, which results in broadly similar steric and hydrophobic interactions within the enzyme binding sites. Thus, the investigated substituents appear to modulate the predicted interactions without producing large changes in the overall calculated binding energies. Importantly, these relatively small differences in docking scores should not be interpreted as quantitative predictors of experimental inhibitory potency, since docking scores provide approximate estimates of ligand–protein interactions and do not fully account for factors such as solvation, protein flexibility, entropy, and kinetic effects. Accordingly, the docking results are used primarily to compare plausible binding modes and interaction patterns rather than to establish a direct ranking of biological potency [17]. Although the present docking analysis provides plausible static binding modes, further molecular dynamics simulations will be required to evaluate the dynamic stability of the predicted ligand-protein complexes.

#### 2.4.2. Protein-Ligand Interaction

##### Tyrosinase-Compound **4** Interaction

**Figure 3:**
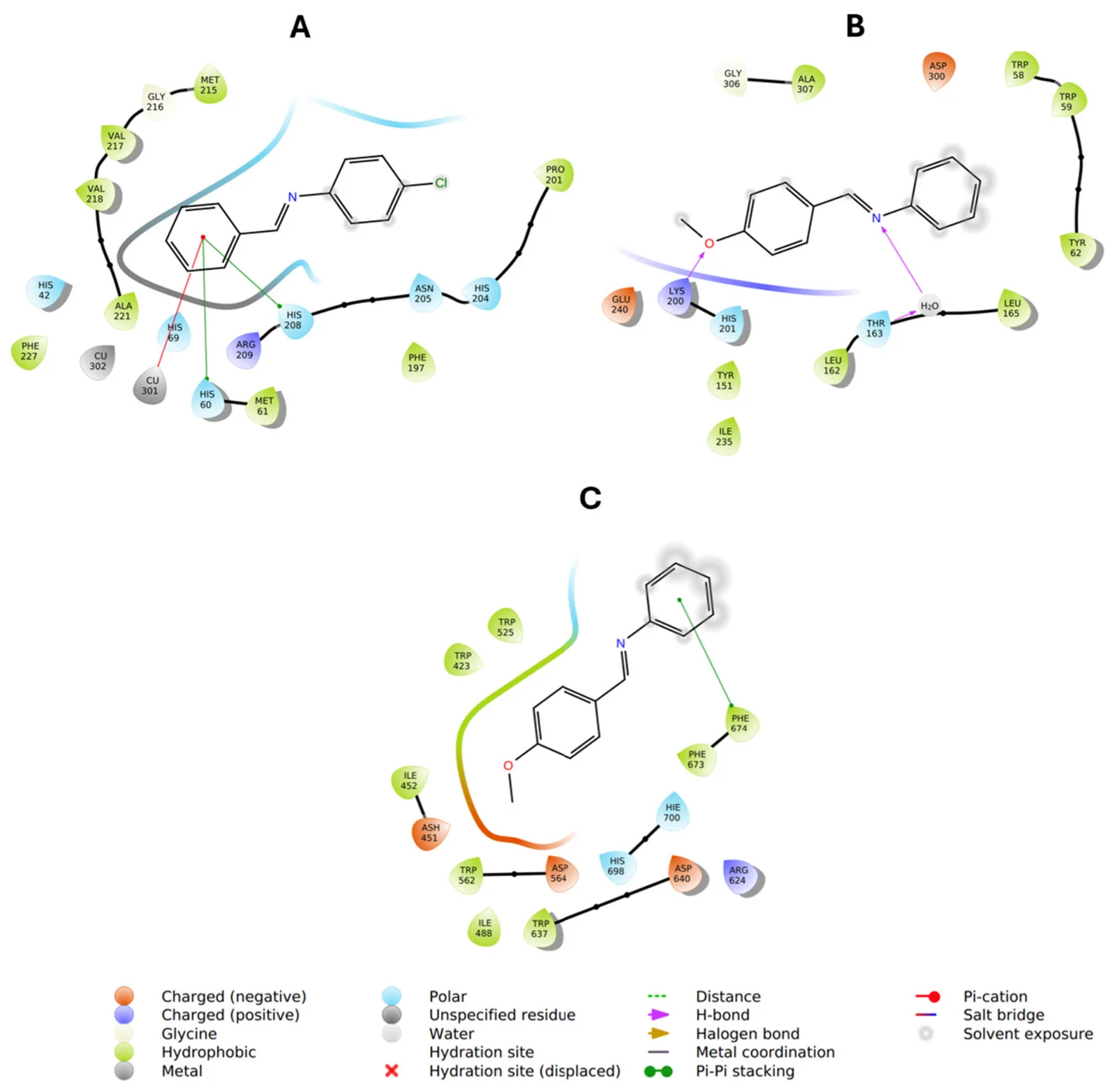
Predicted protein–ligand interaction map: (**A**) tyrosinase-compound **4**, (**B**) α-amylase- compound **2**, and (**C**) α-glucosidase- compound **1**, highlighting key hydrogen bonds, hydrophobic contacts, π–π interactions, and metal-associated contacts (for tyrosinase) with catalytic residues.

The 2D interaction profile of compound **4** within the tyrosinase active site reveals a well-coordinated network of contacts that supports its relatively favorable docking score (Figure 3A). A key feature of the binding mode is the involvement of the binuclear copper center (Cu301 and Cu302) in π-cation interactions, which may suggest interference with tyrosinase catalytic activity. Such interactions are commonly associated with effective tyrosinase inhibitors, as disruption of copper coordination can impair substrate oxidation [18].

In addition to metal coordination, the ligand is stabilized by π–π stacking interactions with His60 and His208, which involve the aromatic ring of compound **4** aligning with the imidazole ring of the histidine residue. This type of interaction enhances binding affinity by contributing favorable van der Waals and electronic interactions within the hydrophobic pocket. Furthermore, His60 and His69, which are known to coordinate copper ions in the active site, also participate indirectly in ligand stabilization [18], reinforcing the positioning of compound **4** near the catalytic core. The presence of hydrophobic residues such as Val217, Val218, Met215, and Ala221 surrounding the ligand indicates that hydrophobic interactions play a significant role in stabilizing the complex. These residues form a nonpolar environment that accommodates the aromatic scaffold of compound **4**, reducing solvent exposure and enhancing binding stability. Additionally, residues like Phe197 and Pro201 contribute to the hydrophobic pocket architecture, further supporting ligand retention within the binding site. Furthermore, van der Waals interactions also contribute to the binding profile. Residues such as Asn205 and His204, and Arg209 are positioned near the ligand and may facilitate weak interactions, although these appear secondary compared to the dominant hydrophobic and metal-coordination contributions [19]. 

### 2.5. DFT Calculations

#### 2.5.1. FMOs Analysis and Global Reactivity Parameters

**Figure 4:**
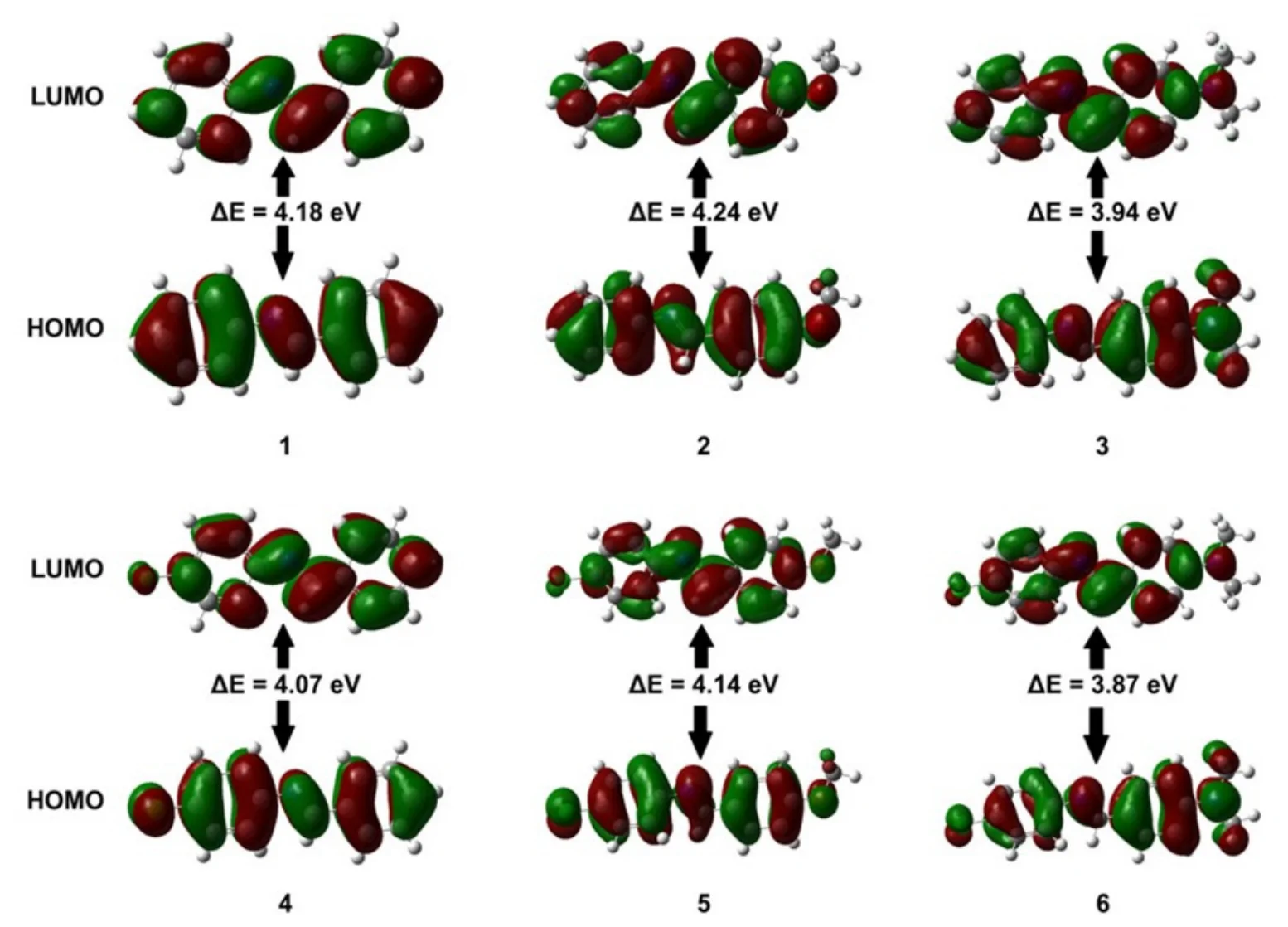
HOMO and LUMO orbital distributions and energy band gaps (ΔE) of compounds **1**–**6**.

The DFT calculations were performed using Gaussian 16 at the B3LYP/6-311++G(d,p) level of theory [20,21,22], as described in Section 3.5. Frequency calculations were subsequently performed at the same B3LYP/6-311++G(d,p) level of theory, and no imaginary frequencies were observed for any of the optimized structures, confirming that they correspond to true minima on the potential energy surface. Frontier Molecular Orbitals (FMOs) are key parameters for understanding the electronic reactivity and chemical stability of molecular systems [23]. The Highest Occupied Molecular Orbital (HOMO) is associated with the electron-donating ability of a molecule, whereas the Lowest Unoccupied Molecular Orbital (LUMO) represents its tendency to accept electrons [24,25]. The energy gap between these frontier orbitals (ΔE) is a significant descriptor of molecular hardness and polarizability, determining the reactivity and interaction capability of the molecule. The HOMO and LUMO orbital distributions of compounds **1**–**6** are illustrated in Figure 4, while orbital energies, calculated band gaps (ΔE) and reactivity parameters are listed in Table S1. However, these frontier molecular orbital descriptors should not be regarded as direct predictors of antioxidant potency, since no quantitative or statistical correlation between the calculated descriptors and the experimental antioxidant activities was established. Furthermore, because the calculations were performed in the gas phase, solvent-dependent effects on the electronic structure, charge distribution, MEP, and frontier orbital energies were not explicitly considered. Therefore, these descriptors should primarily be interpreted as comparative electronic parameters within the investigated series. The orbital density maps (Figure 4) confirm that the HOMO is mainly delocalized over the π-conjugated aromatic framework, whereas the LUMO is distributed over similar regions but exhibits an antibonding character. As summarized in Table S1, the calculated ΔE values for compounds **1**–**6** range from 3.87 to 4.24 eV. The calculated ΔE values for compounds **1**–**6** ranged from 3.87 to 4.24 eV, with compound **6** showing the lowest value (3.87 eV) and compound **2** the highest (4.24 eV). These differences indicate modest substituent-dependent variations in the electronic properties of the investigated compounds. However, given the relatively narrow ΔE range, these differences should not be interpreted as direct predictors of biological potency. Rather, the HOMO-LUMO analysis provides a comparative description of electronic characteristics, including relative charge-transfer propensity, while biological activity is also governed by molecular recognition, steric complementarity, solvation, and specific ligand–target interactions [26,27]. The band gaps obtained for compounds **1**–**6** are therefore in good agreement with the literature. In addition to the FMO analysis, global reactivity descriptors [28] such as ionization potential (I, tendency to lose electrons), electron affinity (A, capacity to accept electrons), electronegativity (χ, tendency to attract electrons), chemical potential (μ, tendency of electrons to escape), chemical hardness (η, resistance to charge transfer), chemical softness (S, inverse of hardness), and electrophilic index (ω, stabilization upon electron acceptance) were calculated from the FMO energies.

As presented in Table S1, the values of χ, η, S and ω were found to be in the ranges of 3.44–4.29 eV, 1.93–2.12 eV, 0.24–0.26 eV^−1^ and 2.99–4.53 eV, respectively. The negative values of μ (−4.29 to −3.44 eV) indicate a predominantly electrophilic character for all compounds. Overall, the global parameters obtained for **1**–**6** are aligned with those reported in the literature for similar π-conjugated compounds [26,27].

#### 2.5.2. Molecular Electrostatic Potential (MEP) Surfaces Analysis

**Figure 5:**
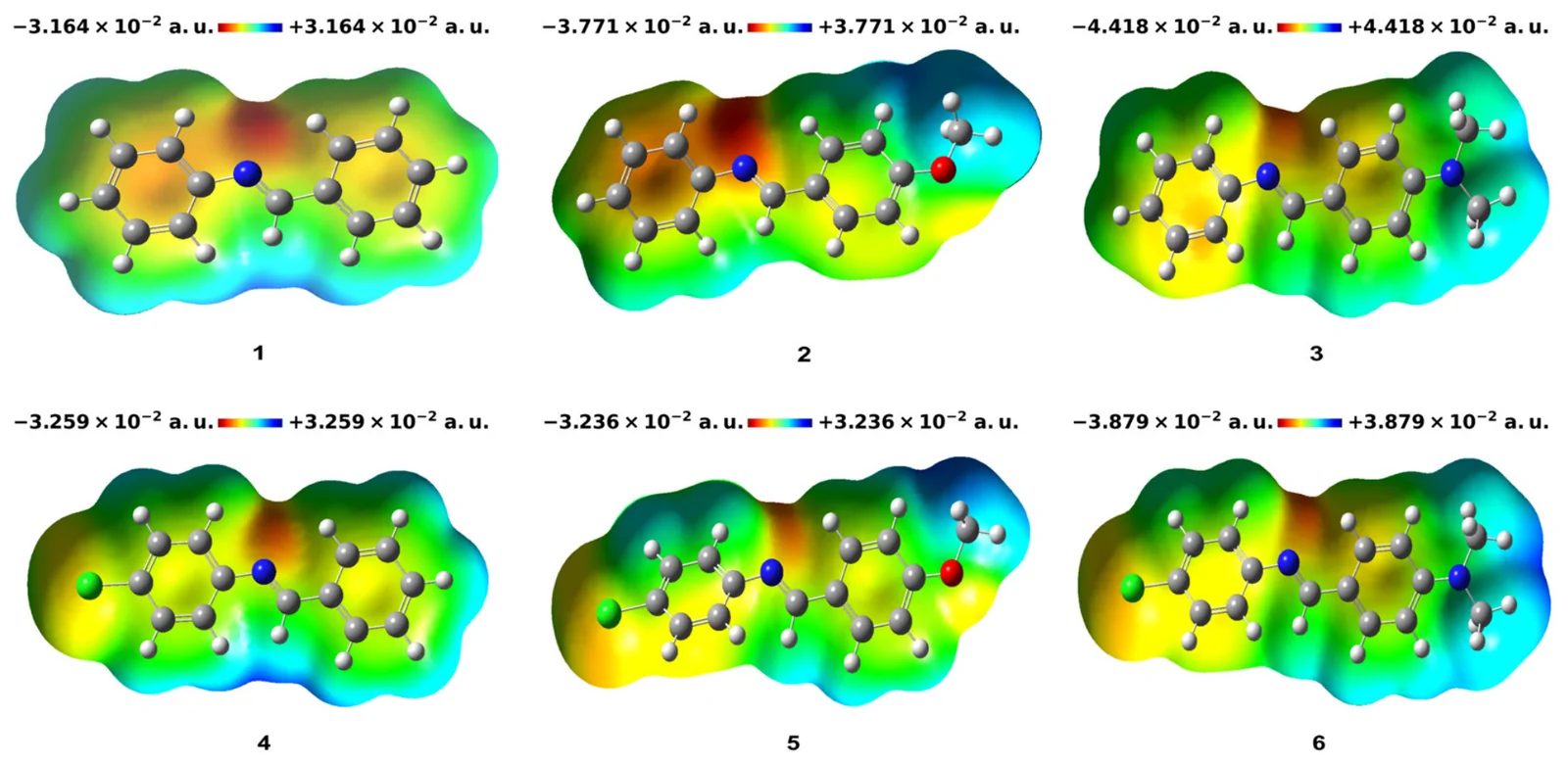
Molecular electrostatic potential (MEP) surface maps of compounds **1**–**6** (red: regions of negative electrostatic potential, blue: regions of positive electrostatic potential).

The molecular electrostatic potential (MEP) surface maps the electrostatic potential onto the molecular electron density, providing insight into the spatial distribution of reactive sites [25]. In these representations, red regions correspond to negative electrostatic potential (nucleophilic sites susceptible to electrophilic attack), whereas blue regions indicate positive electrostatic potential (electrophilic sites favorable for nucleophilic interactions). Intermediate color transitions (orange < yellow < green < blue) indicate increasingly electropositive potential [29]. The calculated MEP potential ranges are **1** (±3.164 × 10^−2^), **2** (±3.771 × 10^−2^), **3** (±4.418 × 10^−2^), **4** (±3.259 × 10^−2^), **5** (±3.236 × 10^−2^), and **6** (±3.879 × 10^−2^) a.u. (Figure 5). Across all six compounds, a pronounced accumulation of negative electrostatic potential is consistently observed around the nitrogen atom of the –N=CH– bridge, originating from the localized lone pair electrons. This persistent nucleophilic region is expected to facilitate hydrogen-bond formation with N–H or O–H donor groups in biological targets, in agreement with the well-established molecular recognition behavior of Schiff base frameworks. 

Within the compound **1**, **2** and **3** series, the MEP profiles evolve systematically with increasing functional group complexity, following the order **1** < **2** < **3**. Compound **1** exhibits the narrowest and most homogeneous potential distribution, consistent with its limited functionalization and reduced electronic polarization. Upon introduction of the methoxy group in compound **2**, a localized region of strongly negative electrostatic potential emerges around the oxygen atom, establishing a second nucleophilic center in addition to the azomethine nitrogen; this dual-site character reflects the pronounced electron-withdrawing nature of the methoxy group. Compound **3** displays the widest MEP range (±4.418 × 10^−2^ a.u.), with charge separation extending across the entire π-conjugated framework, indicating enhanced delocalization and increased electrostatic anisotropy.

The chloro-substituted analogues (**4**, **5**, **6**) follow the same progressive ordering (**4** < **5** < **6**); however, chlorine substitution primarily operates through an inductive electron-withdrawing effect that modulates, rather than overrides, the parent electrostatic profiles. As a result, the surface charge of these compounds is redistributed more uniformly, leading to a partial attenuation of the electrostatic contrast observed for compounds **1**–**3**. In compound **5**, the dual nucleophilic character inherited from compound **2** is largely retained, although the negative potential region becomes more contracted, whereas in compounds **4** and **6**, the aryl chloro substituent promotes electrostatic homogenization of the molecular surface without generating additional localized nucleophilic sites. The MEP analysis complements the FMO results by providing spatial information on the distribution of electron-rich and electron-deficient regions that is not conveyed by the HOMO-LUMO energy gap alone. In particular, the negative electrostatic potential around the azomethine nitrogen and the substituent-dependent variations in surface polarization identify regions that may participate in hydrogen-bonding and electrostatic interactions with biological targets. Therefore, the MEP maps are used here to support the interpretation of the interaction patterns observed in the docking analysis rather than as independent predictors of biological activity. Overall, the DFT and MEP results provide complementary electronic and spatial information for interpreting substituent effects, whereas the experimentally determined biological activities remain the primary evidence for potency.

## 3. Materials and Methods

### 3.1. Materials

Reagents were purchased from Fluka Chemie AG (Buchs) and Sigma-Aldrich and were used as received without additional purification. ^1^H and ^13^C NMR spectra were recorded in DMSO using an Agilent spectrometer operating at 400 MHz. Mass spectral analyses were carried out by ESI-MS (Bruker UltrafleXtreme). FTIR analyses were performed with Shimadzu IRSpirit-X.

Chemical shifts (δ) are reported in parts per million (ppm) relative to the residual solvent signal. For spectra recorded in DMSO-d_6_, the residual solvent peaks at δ 2.50 ppm and δ 39.52 ppm were used as internal references for ^1^H and ^13^C NMR spectra, respectively. Coupling constants (J) are reported in hertz (Hz). Signal multiplicities are designated as s (singlet), d (doublet), dd (doublet of doublets), t (triplet), and m (multiplet).

### 3.2. Synthesis Procedure for Compounds ***1***–***6***

A general synthetic procedure was employed for the preparation of imines **1**–**6**. Aromatic aldehydes (0.02 mol) and the corresponding anilines (0.02 mol) were introduced into a dried round-bottom flask containing dichloromethane and 4 Å molecular sieves (1 g per 1.0 mmol of aldehyde). The reaction mixture was stirred at ambient temperature, and the progress of the reaction was monitored by TLC until completion. Subsequently, the mixture was filtered, and the solvent was removed under reduced pressure to afford the target imines [10].

**Compound 1:** N-benzylideneaniline, C_13_H_11_N
Yield (58%), ^1^H NMR (400 MHz, DMSO-d_6_, δ, ppm): 8.60 (s, 1H, CH=N), 7.93 (d, J = 5.0 Hz, 2H, Ar–H), 7.52 (s, 3H, Ar–H), 7.40 (t, J = 7.6 Hz, 2H, Ar–H), 7.25 (d, J = 7.8 Hz, 3H, Ar–H). ^13^C-NMR, DMSO-d_6_, δ, ppm: 161.1, 151.8, 136.4, 131.9, 129.6, 129.2, 129.1, 126.4. FTIR (ATR, ν, cm^−1^): 3061 (Ar–H), 2892 (CH=N), 1625 (C=N), 1450 (Ar C=C), 1192 (C–N/C–O), 690, 541.
**Compound 2:** N-(4-methoxybenzylidene)aniline, C_14_H_13_NO
Yield (50%), ^1^H NMR (400 MHz, DMSO-d_6_, δ, ppm): 8.50 (s, 1H, CH=N), 7.87 (d, J = 8.7 Hz, 2H, Ar–H), 7.38 (t, J = 7.7 Hz, 2H, Ar–H), 7.20 (d, J = 6.7 Hz, 3H, Ar–H), 7.05 (d, J = 8.6 Hz, 2H, Ar–H), 3.82 (s, 3H, CH_3_). ^13^C-NMR, DMSO-d_6_, δ, ppm:162.3, 160.3, 152.2, 130.9, 129.6, 129.3, 125.9, 121.3, 114.6, 55.8. FTIR (ATR, ν, cm^−1^): 3002 (Ar–H), 2965 (C–H), 1602 (C=N), 1574 (Ar C=C), 1242 (C–N/C–O), 693, 544. ESI-MS m/z: Calculated: 211.26, Found: 212.10 [M+H]^+^
**Compound 3:** N,N-dimethyl-4-((phenylimino)methyl)aniline, C_15_H_16_N_2_
Yield (47%), ^1^H NMR (400 MHz, DMSO-d_6_, δ, ppm): 8.38 (s, 1H, CH=N), 7.72 (d, J = 8.5 Hz, 2H, Ar–H), 7.35 (t, J = 6.7 Hz, 2H, Ar–H), 7.16 (d, J = 7.5 Hz, 3H, Ar–H), 6.76 (d, J = 8.6 Hz, 2H, Ar–H), 2.99 (s, 6H, CH_3_). ^13^C-NMR, DMSO-d_6_, δ, ppm: 160.3, 152.7, 130.7, 129.5, 125.3, 124.2, 121.2, 111.8.
FTIR (ATR, ν, cm^−1^): 3019 (Ar–H), 2963 (C–H), 1602 (C=N), 1599 (Ar C=C), 1249 (C–N/C–O), 838, 539. ESI-MS m/z: Calculated: 224.30, Found: 224.99 [M+H]^+^
**Compound 4:** N-benzylidene-4-chloroaniline, C_13_H_10_ClN
Yield (68%), ^1^H NMR (400 MHz, DMSO-d_6_, δ, ppm): 8.61 (s, 1H, CH=N), 7.92 (d, J = 6.2 Hz, 2H, Ar–H), 7.48 (dd, J = 26.1, 7.7 Hz, 5H, Ar–H), 7.28 (d, J = 8.6 Hz, 2H, Ar–H). ^13^C-NMR, DMSO-d_6_, δ, ppm: 161.9, 150.6, 136.2, 132.1, 130.6, 129.5, 129.3, 129.2, 123.3. FTIR (ATR, ν, cm^−1^): 3061 (Ar–H), 2892 (CH=N), 1625 (C=N), 1450 (Ar C=C), 1192 (C–N/C–O), 690, 541. ESI-MS m/z: Calculated: 215.68, Found: 216.93 [M+H]^+^
**Compound 5:** 4-chloro-N-(4-methoxybenzylidene)aniline, C_14_H_12_ClNO
Yield (60%), ^1^H NMR (400 MHz, DMSO-d_6_, δ, ppm): 8.51 (s, 1H, CH=N), 7.86 (d, J = 8.7 Hz, 2H, Ar–H), 7.42 (d, J = 8.6 Hz, 2H, Ar–H), 7.23 (d, J = 8.6 Hz, 2H, Ar–H), 7.05 (d, J = 8.7 Hz, 2H, Ar–H), 3.81 (s, 3H, CH_3_). ^13^C-NMR, DMSO-d_6_, δ, ppm: 162.5, 161.0, 150.9, 148.1, 132.2, 130.18 (s), 129.5, 128.9, 123.2, 115.5, 114.7, 55.8. FTIR (ATR, ν, cm^−1^): 3019 (Ar–H), 2963 (C–H), 1602 (C=N), 1599 (Ar C=C), 1249 (C–N/C–O), 838, 538. ESI-MS m/z: Calculated: 245.70, Found: 245.80 [M]^+^
**Compound 6:** 4-(((4-chlorophenyl)imino)methyl)-N,N-dimethylaniline, C_15_H_15_ClN_2_
Yield (56%), ^1^H NMR (400 MHz, DMSO-d_6_, δ, ppm): 8.39 (s, 1H, CH=N), 7.72 (d, J = 8.6 Hz, 2H, Ar–H), 7.39 (d, J = 8.4 Hz, 2H, Ar–H), 7.19 (d, J = 8.4 Hz, 2H, Ar–H), 6.76 (d, J = 8.6 Hz, 2H, Ar–H), 3.00 (s, 6H, CH_3_). ^13^C-NMR, DMSO-d_6_, δ, ppm: 161.1, 130.8, 129.4, 128.9, 123.1, 115.5, 111.8, 111.5.
FTIR (ATR, ν, cm^−1^): 3001 (Ar–H), 2853 (C–H), 1601 (C=N), 1598 (Ar C=C), 1162 (C–N/C–O), 815, 534. ESI-MS m/z: Calculated: 258.75; Found: 258.89 [M]^+^

### 3.3. Antimicrobial Activity

Test Microorganisms: In the study, S. aureus ATCC 29213, S. epidermidis ATCC 12228, E. faecalis ATCC 29212, E. coli ATCC 25922, A. baumannii ATCC 19606, P. aeruginosa ATCC 27853, C. parapsilosis ATCC 22019 and C. albicans ATCC 10231 were used. The microorganism cultures (The American Type Culture Collection (ATCC) standard strains) utilized in the study were obtained from the culture collection of the Department of Pharmaceutical Microbiology, Faculty of Pharmacy, University of Health Sciences. One day prior to the experiments, the microorganisms were activated (Tryptic Soy Agar for bacterial strains and Sabouraud Dextrose Agar for yeast strains). Colonies obtained from the activated cultures were suspended in Mueller–Hinton Broth (MHB), adjusted to a turbidity equivalent to the 0.5 McFarland standard (10^8^ CFU/mL), and then diluted with medium to a final concentration of 10^6^ CFU/mL [30].

*Determination of Minimum inhibitory concentration (MIC):*

The minimum inhibitory concentration (MIC) of the tested components was evaluated using the microdilution technique in accordance with Clinical and Laboratory Standards Institute (CLSI) guidelines [30]. Serial two-fold dilutions were prepared in suitable media, namely MHB for bacterial strains and RPMI for Candida species. For the compounds, concentrations ranged from 2000 µg/mL to 1.95 µg/mL using an automatic pipetting system. Tigecycline and voriconazole served as positive controls and were tested across a concentration range of 512–0.5 µg/mL. Following incubation for 24 h (37 °C for bacteria and 27 °C for yeasts), 50 µL of 2 mg/mL 2,3,5-triphenyltetrazolium chloride (TTC, Merck) was added to each well. MIC endpoints were determined after 30 min based on observable color changes. DMSO were included as negative controls, and all assays were conducted in triplicate to ensure reproducibility.

### 3.4. Enzyme Inhibition and Antioxidant Activity Studies

In vitro *Antioxidant Assays*

To assess the antioxidant potential of the compounds, six complementary in vitro spectrophotometric assays were performed. DPPH, ABTS, CUPRAC, and FRAP assays were performed according to previously reported methods [DPPH [31], ABTS [32], CUPRAC [33], FRAP [34]], respectively. Metal-chelating activity (MCA) and phosphomolybdenum (PBD) assays were performed following the previously published methodology [31]. The antioxidant activities were expressed as IC_50_ values (mg/mL), as reported in Table 2. Trolox and EDTA were used as reference standards.

*Enzyme inhibitory activity*

The enzyme inhibitory assays were performed as earlier reports [31]. For the tyrosinase inhibition assay, the sample solution (25 µL) was mixed with tyrosinase solution (40 µL, Sigma) and phosphate buffer (100 µL, pH 6.8) in a 96-well microplate and pre-incubated for 15 min at 25 °C. The reaction was initiated by adding L-DOPA (40 µL), followed by incubation for 10 min at 25 °C, and the absorbance was measured at 492 nm.

For the α-amylase inhibition assay, the sample solution (25 µL) was mixed with α-amylase solution (50 µL; from porcine pancreas, EC 3.2.1.1, Sigma) in phosphate buffer (pH 6.9 containing 6 mM NaCl) and pre-incubated for 10 min at 37 °C. The reaction was initiated by adding starch solution (50 µL, 0.05%) and incubated for a further 10 min at 37 °C. The reaction was stopped by adding HCl (25 µL, 1 M), followed by iodine–potassium iodide solution (100 µL), and the absorbance was measured at 630 nm.

For the α-glucosidase inhibition assay, the sample solution (50 µL) was mixed with glutathione (50 µL), α-glucosidase solution (50 µL; from Saccharomyces cerevisiae, EC 3.2.1.20, Sigma), phosphate buffer (pH 6.8), and PNPG (4-nitrophenyl-α-D-glucopyranoside; 50 µL) in a 96-well microplate. After incubation for 15 min at 37 °C, the reaction was stopped by adding sodium carbonate (50 µL, 0.2 M), and the absorbance was measured at 400 nm.

Appropriate blanks without enzyme were prepared for each assay, and blank absorbance was subtracted from the corresponding sample absorbance. All assays were performed in triplicate, and the results were expressed as IC_50_ values. Acarbose was used as the reference inhibitor for α-amylase and α-glucosidase, while kojic acid was used for tyrosinase. 

*DFT Calculations*

### 3.5. Molecular Docking and DFT Calculations

The X-ray crystal structures for tyrosinase (PDB ID: 6QXD) [35], α-amylase (PDB ID: 1B2Y) [36], and α-glucosidase (PDB ID: 7KBJ) [37] were retrieved from the Protein Data Bank (PDB). This target selection enabled the computational analysis to complement the experimental assessment of tyrosinase, α-amylase, and α-glucosidase inhibitory activities by examining the potential binding modes and molecular interactions of the investigated compounds with the same enzyme targets used in the in vitro assays. Protein structures were initially preprocessed using the PrepareProtein program, which is available on the PlayMolecule web server [38]. During this stage, hydrogen atoms that were not present in the crystal structures were added, and the protonation states of titratable amino acid residues were assigned using predicted pKa values to approximate physiological conditions (pH 7.4). The 3D ligand structures were obtained from the PubChem database. Each ligand structure was geometrically optimized by conducting energy reduction in UCSF Chimera to obtain energetically stable conformations [39].

The prepared protein and ligand files were processed using MGLTools (version 1.5.6) [40], which merged nonpolar hydrogen atoms and assigned Gasteiger partial charges. The docking grid boxes for tyrosinase (X: 29.99, Y: 18.21, Z: 96.45 Å), α-amylase (X: −1.54, Y: −44.04, Z: 22.63 Å), and α-glucosidase (X: −13.77, Y: 24.04, Z: 12.35 Å) were defined as 40 × 40 × 40 Å to cover the whole active site [41]. The grid boxes were selected to encompass the experimentally characterized active site of each enzyme while providing sufficient spatial coverage for the investigated compounds to explore the relevant binding pocket without imposing an unnecessarily restrictive search space. The grid dimensions were kept consistent across the three targets to ensure a comparable docking setup, while the grid centers were individually defined according to the location of the corresponding experimentally occupied ligand-binding regions. The Lamarckian genetic approach implemented in AutoDock 4.2.6 was used for ligand conformational sampling, with 10 independent docking calculations for each ligand-protein combination [40]. The resulting poses were examined to assess the consistency of the predicted binding modes, rather than interpreting the numerical score of a single pose in isolation. Alternative protonation/tautomeric states were not independently docked as separate ligand states. The top poses were then analyzed for protein-ligand interactions and displayed with Maestro Viewer. The reliability of the docking protocol was assessed by re-docking the experimentally co-crystallized ligands into the active sites of the corresponding enzymes. As illustrated in Figure S1 the re-docked poses closely reproduced the experimentally determined ligand orientations in tyrosinase, α-amylase, and α-glucosidase. The agreement between the experimental and predicted poses indicates that the docking procedure successfully identified the relevant binding regions and reproduced the characteristic ligand conformations within the respective catalytic pockets. The heavy-atom RMSD values between the crystallographic and re-docked ligand poses were 1.15 Å for tyrosinase, 1.21 Å for α-amylase, and 1.66 Å for α-glucosidase. All values were below the commonly applied 2.0 Å threshold for acceptable pose reproduction, supporting the suitability of the docking protocols for subsequent analysis of the investigated compounds [42,43].

Quantum chemical calculations for the synthesized Schiff base derivatives (**1**–**6**) were performed using Density Functional Theory (DFT) with the Gaussian 16 (Revision C.01) software package [20]. The ground state geometries were optimized in the gas phase at the B3LYP/6-311++G(d,p) level of theory [21,22]. To determine the electronic properties and reactive regions of the molecules, Frontier Molecular Orbitals (HOMO–LUMO) and Molecular Electrostatic Potential (MEP) surfaces were computed. GaussView 6.1 was used to visualize the orbital distributions and MEP maps.

## 4. Conclusions

In this study, a series of six benzylidene-based Schiff base derivatives bearing electron-donating and electron-withdrawing substituents were successfully synthesized and characterized by FT-IR, ^1^H NMR, ^13^ C NMR, and ESI-MS analyses. The biological evaluations revealed that the nature of the aromatic substituents influenced the antioxidant, enzyme inhibitory, and antimicrobial properties of the synthesized compounds. Compounds **3** and **6** exhibited the most promising antioxidant activities, particularly in the ABTS, CUPRAC, FRAP, phosphomolybdenum, and metal-chelating assays, highlighting the favorable contribution of the dimethylamino substituent to the antioxidant properties of this series. In the enzyme inhibition studies, compounds **1** and **2** showed the highest tyrosinase inhibitory activities within the synthesized series. For α-glucosidase inhibition, compounds **1**–**3** and **5** exhibited measurable activity, with compound **5** showing the highest activity among the synthesized derivatives. However, comparison of the IC_50_ values on a molar basis demonstrated that all active synthesized compounds were less potent than acarbose. These findings indicate that structural and electronic modification of the Schiff base framework influences enzyme inhibitory activity, although further optimization is required to improve potency. Antimicrobial investigations revealed moderate activity across the series, with compound **3** showing a comparatively favorable antibacterial profile, particularly against *E. coli* and *E. faecalis*. Molecular docking studies provided plausible binding modes and identified interactions of the compounds with residues within the active sites of tyrosinase, α-amylase, and α-glucosidase. DFT calculations further demonstrated substituent-dependent variations in frontier molecular orbital characteristics and electrostatic potential distributions. However, the relatively small differences in HOMO–LUMO energy gaps across the series should not be considered direct predictors of biological potency. Furthermore, because the calculations were performed in the gas phase, the calculated electronic descriptors do not explicitly account for solvent and buffer effects present under the experimental conditions and should therefore be interpreted primarily as comparative electronic parameters. Instead, the DFT and MEP analyses provide complementary electronic and spatial information that supports the interpretation of substituent effects and the interaction patterns observed in the docking studies. To sum up, the combined experimental and computational findings demonstrate that structural and electronic modification of the Schiff base scaffold can modulate its biological properties. Compound **3** exhibited a particularly interesting multifunctional profile, combining strong antioxidant activity within the series with antimicrobial and selected enzyme inhibitory activities, and therefore represents a candidate for further biological and medicinal chemistry investigation. These findings provide useful structure–activity relationship insights and may support the future design and optimization of Schiff base-derived bioactive molecules.

## Figures and Tables

**Table 1:** Chemical structures of synthesized Schiff base compounds (**1**–**6**).

Compound	Chemical Structure
**1**	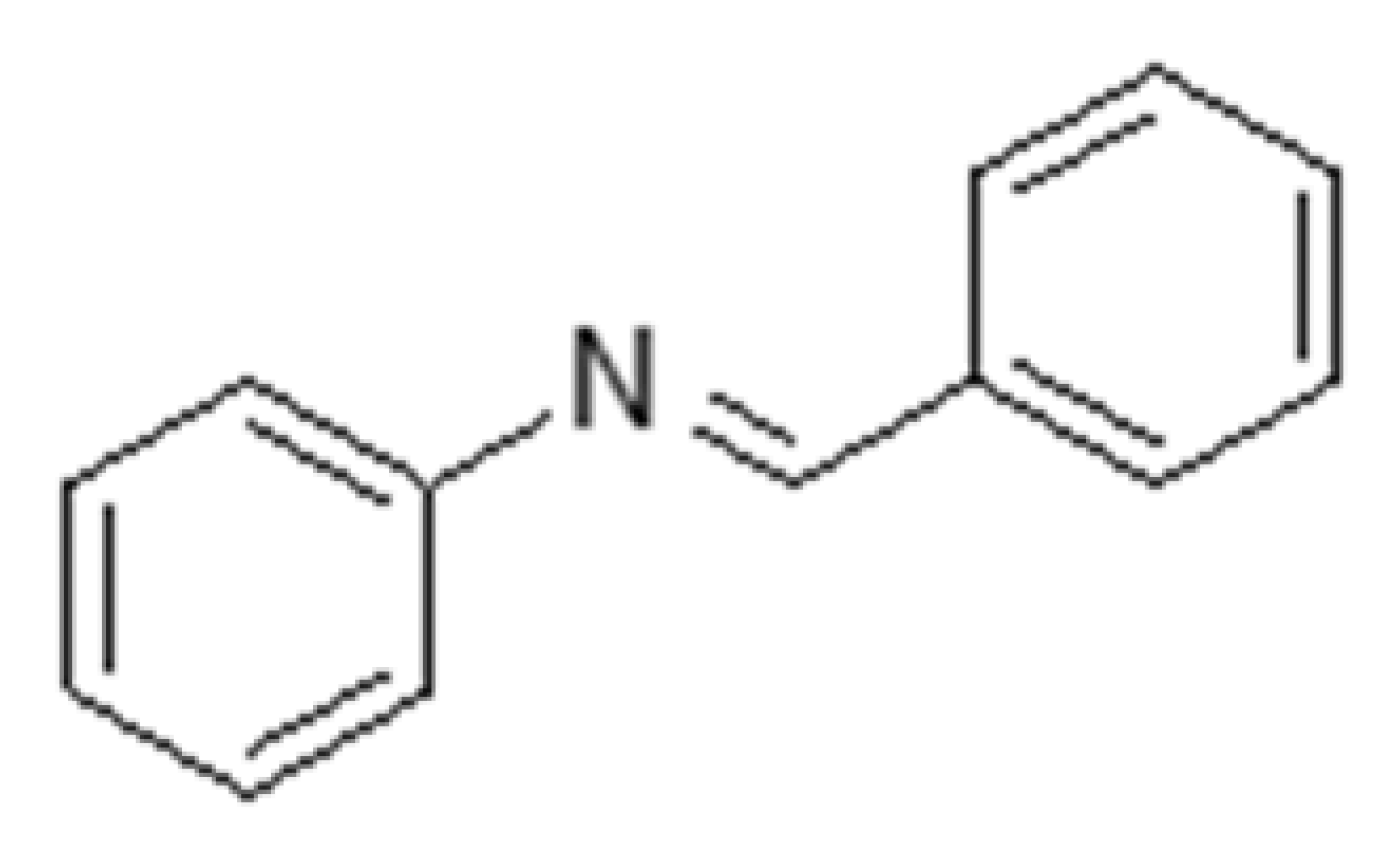
**2**	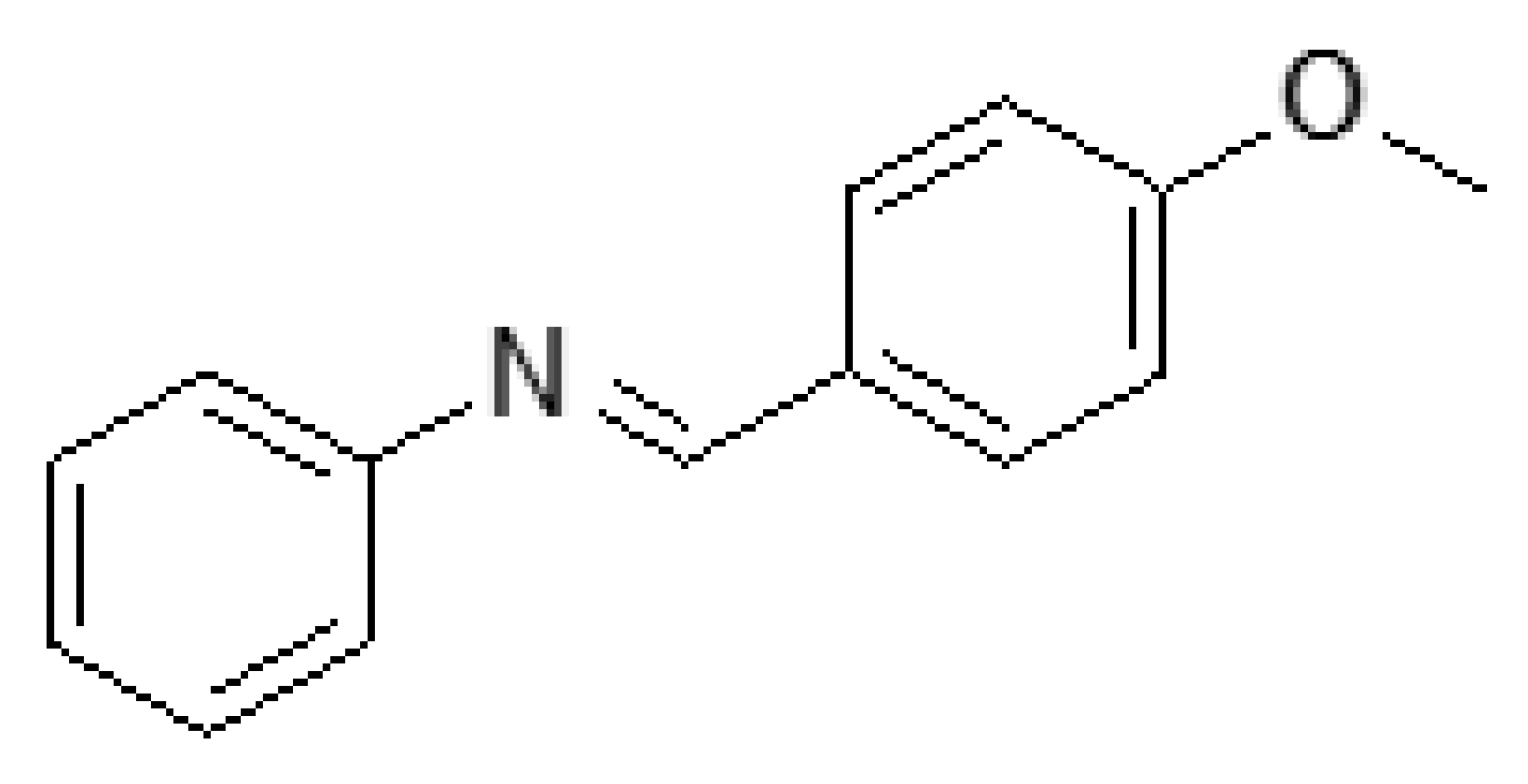
**3**	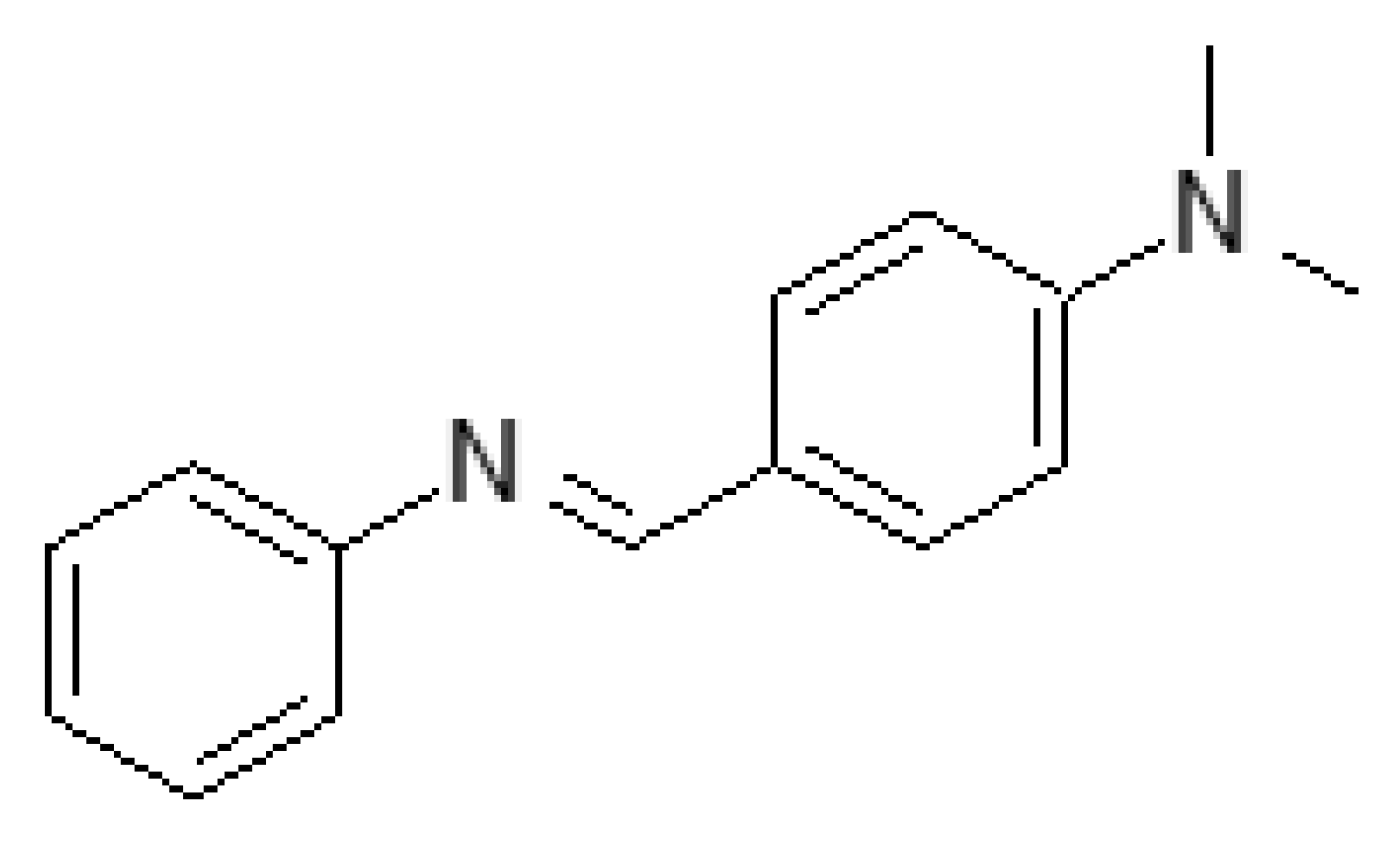
**4**	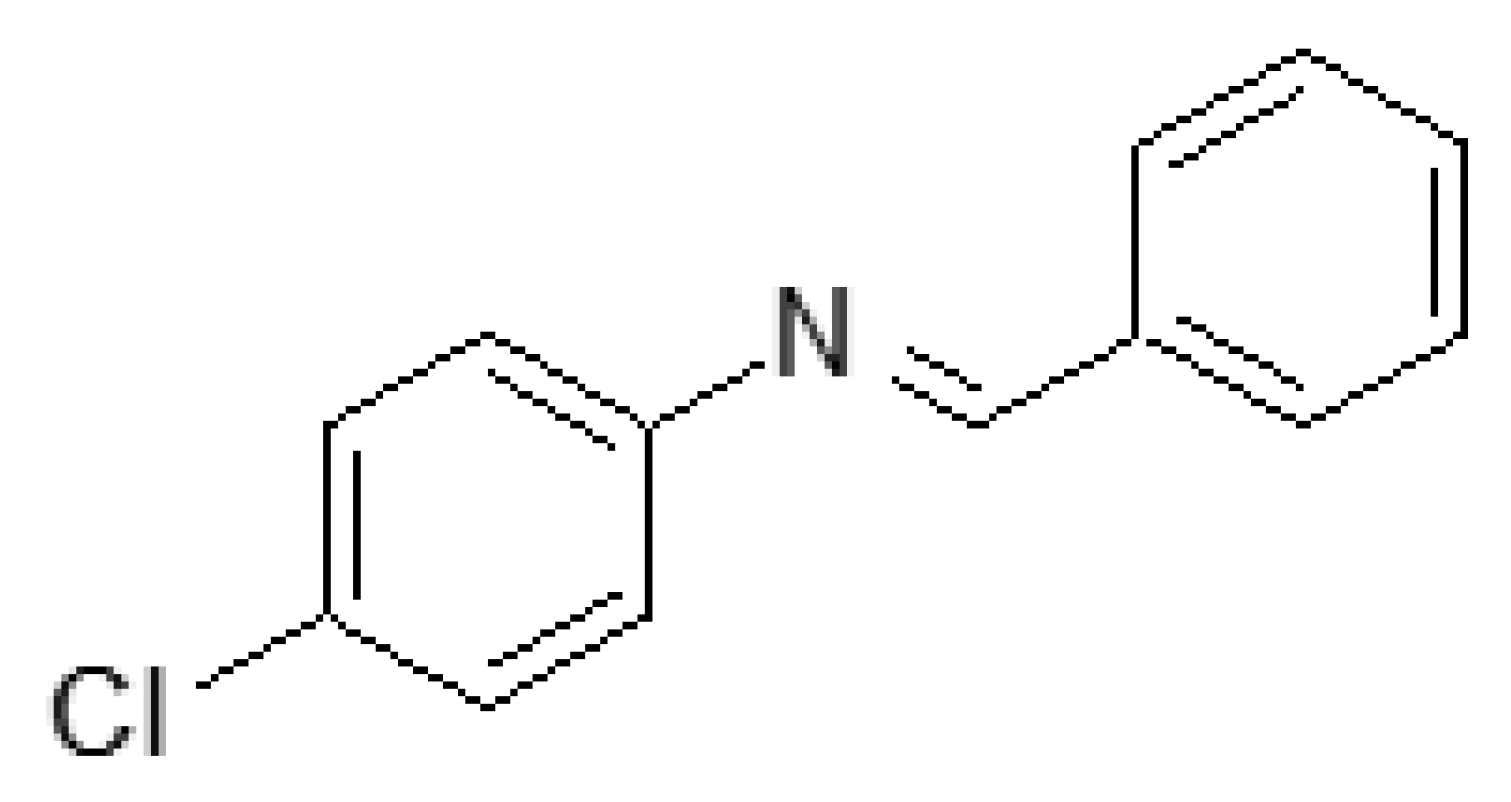
**5**	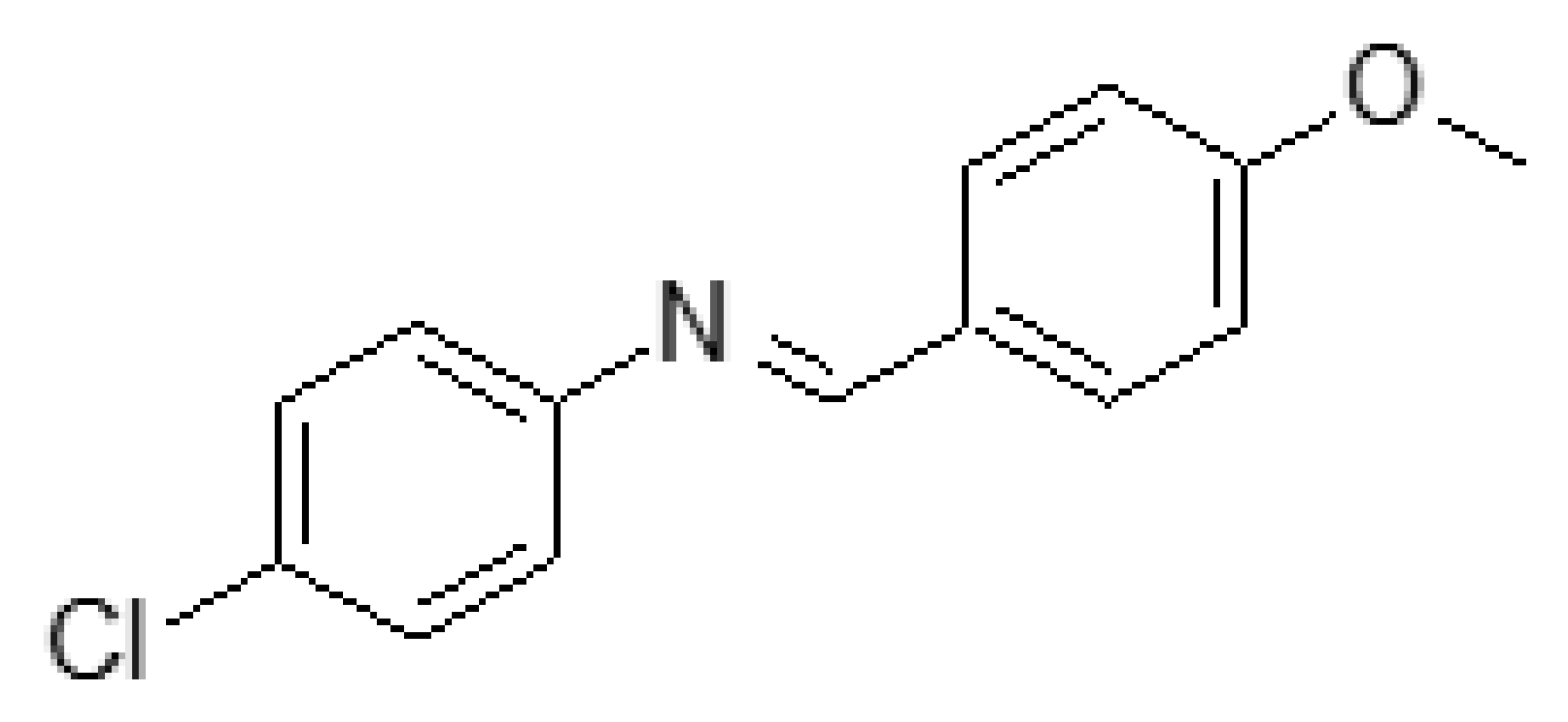
**6**	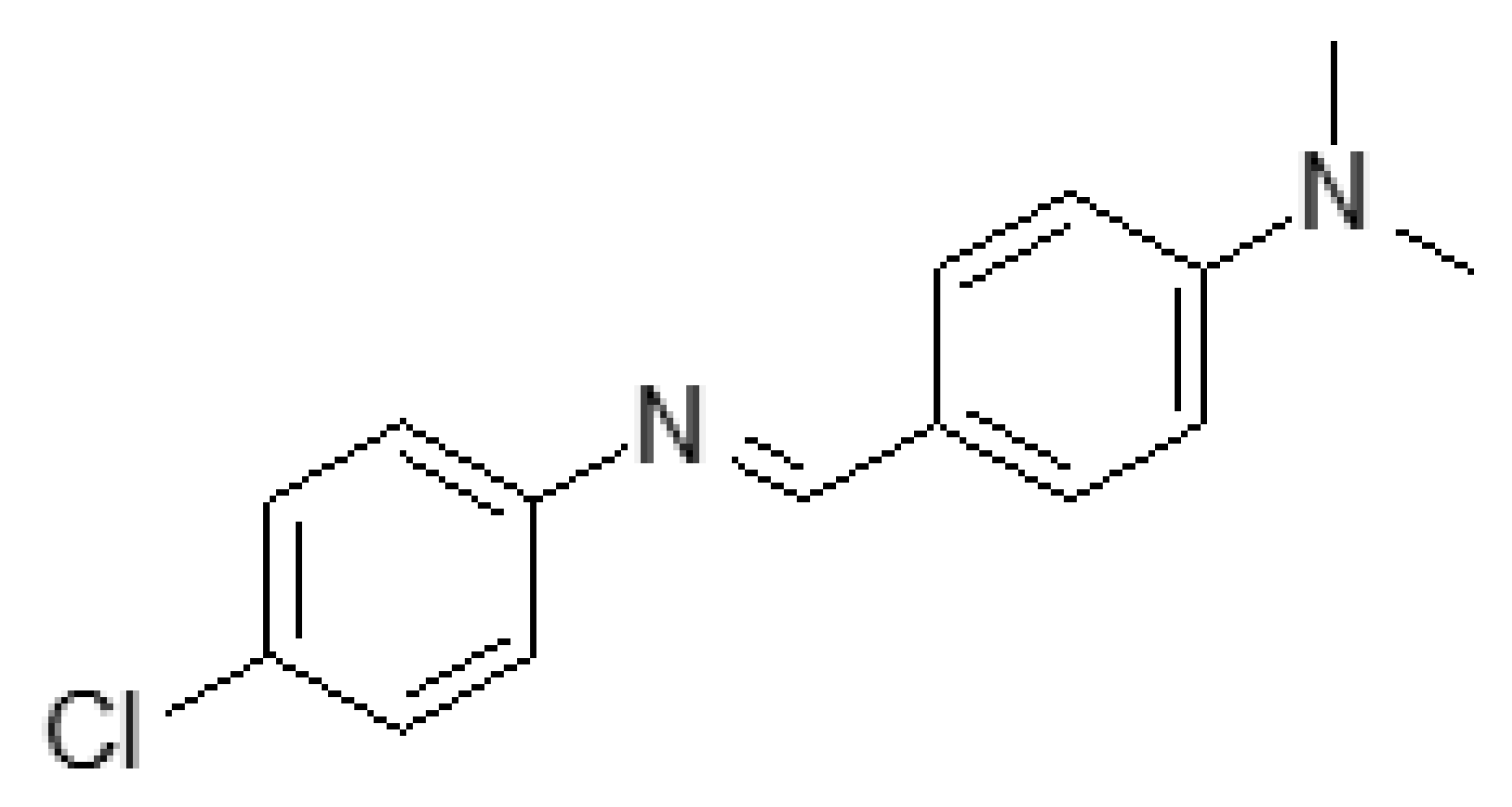

**Table 2:** IC_50_ (mg/mL) values for antioxidant test results of compounds **1**–**6**.

Compound	DPPH	ABTS	CUPRAC	FRAP	Phosphomolybdenum	Chelating
**1**	na	4.45 ± 0.07	4.90 ± 0.02	2.35 ± 0.10	>5	>5
**2**	na	4.69 ± 0.06	>5	2.70 ± 0.08	>5	4.88 ± 1.00
**3**	na	2.20 ± 0.06	1.32 ± 0.04	1.98 ± 0.02	1.83 ± 0.01	2.50 ± 0.03
**4**	na	>5	>5	2.48 ± 0.03	>5	4.07 ± 0.51
**5**	>5	>5	>5	2.44 ± 0.01	>5	>5
**6**	na	2.35 ± 0.06	1.90 ± 0.02	1.67 ± 0.03	1.14 ± 0.14	0.72 ± 0.02
Trolox	0.13 ± 0.01	0.18 ± 0.01	0.12 ± 0.01	0.05 ± 0.01	0.48 ± 0.01	-
EDTA	-	-	-	-	-	0.03 ± 0.001

na: not active.

**Table 3:** Enzyme inhibitory activities of compounds **1**–**6** against tyrosinase, α-amylase, and α-glucosidase enzymes expressed as IC_50_ (mM).

Compound	Tyrosinase	α-Amylase	α-Glucosidase
**1**	8.44 ± 0.06	7.06 ± 0.22	5.57 ± 0.06
**2**	7.76 ± 0.14	6.25 ± 0.05	4.88 ± 0.05
**3**	11.19 ± 0.40	na	4.50 ± 0.04
**4**	na	5.89 ± 0.14	12.61 ± 0.65
**5**	13.72 ± 2.40	6.23 ± 1.38	4.31 ± 0.04
**6**	na	na	na
Kojic acid	0.49 ± 0.01	-	-
Acarbose	-	0.94 ± 0.02	1.70 ± 0.11

na: not active.

**Table 4:** Antimicrobial activity results of compounds **1**–**6** (MIC, μg/mL).

Compounds MIC (µg/mL)							
Microorganisms	1	2	3	4	5	6	Tigecycline/ Voriconazol
**Gram-Positive Bacteria**							
*S. aureus* ATCC 29213	1000	1000	500	500	1000	1000	0.5
*S. epidermidis* ATCC 12228	1000	1000	1000	1000	1000	1000	1
*E. faecalis* ATCC 29212	500	500	250	500	500	500	2
**Gram-Negative Bacteria**							
*E. coli* ATCC 25922	500	500	250	500	500	500	0.5
*A. baumannii* ATCC 19606	500	500	500	500	500	500	16
*P. aeruginosa* ATCC 27853	500	500	500	500	500	500	2
**Yeast**							
*C. albicans* ATCC 10231	1000	1000	500	500	500	500	4
*C. parapsilosis* ATCC 22019	1000	1000	500	500	500	500	4

MIC determinations were performed in three independent experiments (n = 3), and the reported MIC values represent the mean of the three replicate determinations.

**Table 5:** Docking scores (kcal/mol) of the compounds **1**–**6** against tyrosinase, α-amylase, and α-glucosidase.

Compound	Docking Score (kcal/mol)		
	Tyrosinase	α-Amylase	α-Glucosidase
**1**	−5.90	−5.68	−5.76
**2**	−5.95	−5.73	−5.75
**3**	−4.18	−5.26	−5.60
**4**	−6.50	−5.06	−5.68
**5**	−4.07	−5.43	−5.73
**6**	−4.02	−5.12	−4.41

## Data Availability

The original contributions presented in this study are included in the article and Supplementary Materials. Further inquiries can be directed to the corresponding author.

## Supplementary Materials

The following supporting information can be downloaded at: https://www.mdpi.com/article/10.3390/molecules31193519/s1.

## References

[1] Kajal A., Bala S., Kamboj S., Sharma N., Saini V. (2013). Schiff Bases: A Versatile Pharmacophore. J. Catal..

[2] Thakur S., Jaryal A., Bhalla A. (2024). Recent Advances in Biological and Medicinal Profile of Schiff Bases and Their Metal Complexes: An Updated Version (2018–2023). Results Chem..

[3] Mushtaq I., Ahmad M., Saleem M., Ahmed A. (2024). Pharmaceutical Significance of Schiff Bases: An Overview. Futur. J. Pharm. Sci..

[4] Giacomini E., Rupiani S., Guidotti L., Recanatini M., Roberti M. (2016). The Use of Stilbene Scaffold in Medicinal Chemistry and Multi- Target Drug Design. Curr. Med. Chem..

[5] De Filippis B., Ammazzalorso A., Amoroso R., Giampietro L. (2019). Stilbene Derivatives as New Perspective in Antifungal Medicinal Chemistry. Drug Dev. Res..

[6] Zhang A.J., Rimando A.M., Mizuno C.S., Mathews S.T. (2017). α-Glucosidase Inhibitory Effect of Resveratrol and Piceatannol. J. Nutr. Biochem..

[7] Bae S.J., Ha Y.M., Park Y.J., Park J.Y., Song Y.M., Ha T.K., Chun P., Moon H.R., Chung H.Y. (2012). Design, Synthesis, and Evaluation of (E)-N-Substituted Benzylidene-Aniline Derivatives as Tyrosinase Inhibitors. Eur. J. Med. Chem..

[8] Özkay Y., Incesu Z., Işikdaǧ I., Yeşilkaya M. (2008). Antiproliferative Effects of Some N-Benzylideneanilines. Cell Biochem. Funct..

[9] Han S.Y., Inoue H., Terada T., Kamoda S., Saburi Y., Sekimata K., Saito T., Kobayashi M., Shinozaki K., Yoshida S. (2003). N-Benzylideneaniline and N-Benzylaniline Are Potent Inhibitors of Lignostilbene-α, β-Dioxygenase, a Key Enzyme in Oxidative Cleavage of the Central Double Bond of Lignostilbene. J. Enzym. Inhib. Med. Chem..

[10] Wang H., Wang C., Huang K., Liu L., Chang W., Li J. (2016). Copper-Catalyzed Cascade Reaction via Intramolecular Hydroamination Cyclization of Homopropargylic Amines and Intermolecular Povarov Reaction with Imines. Org. Lett..

[11] Uba A.I. (2026). Integrated Computer-Aided Drug Design: Advances in GPCR Natural Ligand Discovery. Cell Biochem. Biophys..

[12] Ferreira L.G., Dos Santos R.N., Oliva G., Andricopulo A.D. (2015). Molecular Docking and Structure-Based Drug Design Strategies. Molecules.

[13] Du X., Li Y., Xia Y.L., Ai S.M., Liang J., Sang P., Ji X.L., Liu S.Q. (2016). Insights into Protein–Ligand Interactions: Mechanisms, Models, and Methods. Int. J. Mol. Sci..

[14] Uba A.I., Zengin G., Montesano D., Cakilcioglu U., Selvi S., Ulusan M.D., Caprioli G., Sagratini G., Angeloni S., Jugreet S. (2022). Antioxidant and Enzyme Inhibitory Properties, and HPLC–MS/MS Profiles of Different Extracts of Arabis Carduchorum Boiss.: An Endemic Plant to Turkey. Appl. Sci..

[15] Zolghadri S., Bahrami A., Hassan Khan M.T., Munoz-Munoz J., Garcia-Molina F., Garcia-Canovas F., Saboury A.A. (2019). A Comprehensive Review on Tyrosinase Inhibitors. J. Enzym. Inhib. Med. Chem..

[16] Etsassala N.G.E.R., Badmus J.A., Marnewick J.L., Iwuoha E.I., Nchu F., Hussein A.A., Egieyeh S. (2022). Alpha-Glucosidase and Alpha-Amylase Inhibitory Activities, Molecular Docking, and Antioxidant Capacities of Plectranthus Ecklonii Constituents. Antioxidants.

[17] Chakraborty K., Saha P., Sil S.K. (2022). Molecular Docking of Natural Compounds from Fenugreek Plant (Trigonella Foenum-Graecum L.) for Investigating the Potential Inhibition of Alpha-Amylase and Alpha-Glucosidase of Type 2 Diabetes. Trends Carbohydr. Res..

[18] Fujieda N., Yabuta S., Ikeda T., Oyama T., Muraki N., Kurisu G., Itoh S. (2013). Crystal Structures of Copper-Depleted and Copper-Bound Fungal pro-Tyrosinase: Insights into Endogenous Cysteine-Dependent Copper Incorporation. J. Biol. Chem..

[19] Barratt E., Bingham R.J., Warner D.J., Laughton C.A., Phillips S.E.V., Homans S.W. (2005). Van Der Waals Interactions Dominate Ligand−Protein Association in a Protein Binding Site Occluded from Solvent Water. J. Am. Chem. Soc..

[20] Frisch M.J., Trucks G.W., Schlegel H.B., Scuseria G.E., Robb M.A., Cheeseman J.R., Scalmani G., Barone V., Petersson G.A., Nakatsuji H. (2016). G16_B01 2016, Gaussian 16, Revision B.01.

[21] Lee C., Yang W., Parr R.G. (1988). Development of the Colle-Salvetti Correlation-Energy Formula into a Functional of the Electron Density. Phys. Rev. B.

[22] Becke A.D. (1988). Density-Functional Exchange-Energy Approximation with Correct Asymptotic Behavior. Phys. Rev. A.

[23] Sagdinc S., Pir H. (2009). Spectroscopic and DFT Studies of Flurbiprofen as Dimer and Its Cu(II) and Hg(II) Complexes. Spectrochim. Acta A Mol. Biomol. Spectrosc..

[24] Tamer Ö., Avcı D., Atalay Y. (2014). Quantum Chemical Characterization of N-(2-Hydroxybenzylidene)Acetohydrazide (HBAH): A Detailed Vibrational and NLO Analysis. Spectrochim. Acta A Mol. Biomol. Spectrosc..

[25] Okur M., Albayrak N., Tamer Ö., Avcı D., Atalay Y. (2018). A Theoretical Study on N′-[(Z)-(4-Methylphenyl)Methylidene] -4-Nitrobenzohydrazide (NMPMN). Braz. J. Phys..

[26] Priyambada Biswal S., Hota P., Ranjan Dash M., Kumari Misra P. (2024). Combined Experimental and Theoretical Investigation of the Electrochemical Behavior of Hydroxy-Substituted Anils. ChemistrySelect.

[27] Hota P., Biswal S.P., Dash M.R., Das B.R., Misra P.K. (2024). Exploring Spectral and Electrochemical Behavior of Hydroxy-N-Benzylideneanilines by Integrated Theoretical and Experimental Approaches. J. Phys. Org. Chem..

[28] Geerlings P., De Proft F., Langenaeker W. (2003). Conceptual Density Functional Theory. Chem. Rev..

[29] Politzer P., Murray J.S. (2002). The Fundamental Nature and Role of the Electrostatic Potential in Atoms and Molecules. Theor. Chem. Acc..

[30] (2023). Performance Standards for Antimicrobial Susceptibility Testing.

[31] Uysal S., Zengin G., Locatelli M., Bahadori M.B., Mocan A., Bellagamba G., De Luca E., Mollica A., Aktumsek A. (2017). Cytotoxic and Enzyme Inhibitory Potential of Two Potentilla Species (*P. speciosa* L. and *P. reptans* Willd.) and Their Chemical Composition. Front. Pharmacol..

[32] Re R., Pellegrini N., Proteggente A., Pannala A., Yang M., Rice-Evans C. (1999). Antioxidant Activity Applying an Improved ABTS Radical Cation Decolorization Assay. Free Radic. Biol. Med..

[33] Apak R., Güçlü K., Özyürek M., Esin Karademir S., Erçağ E. (2006). The Cupric Ion Reducing Antioxidant Capacity and Polyphenolic Content of Some Herbal Teas. Int. J. Food Sci. Nutr..

[34] Aktumsek A., Zengin G., Guler G.O., Cakmak Y.S., Duran A. (2013). Antioxidant Potentials and Anticholinesterase Activities of Methanolic and Aqueous Extracts of Three *Endemic centaurea* L. Species. Food Chem. Toxicol..

[35] Ielo L., Deri B., Germanò M.P., Vittorio S., Mirabile S., Gitto R., Rapisarda A., Ronsisvalle S., Floris S., Pazy Y. (2019). Exploiting the 1-(4-Fluorobenzyl)Piperazine Fragment for the Development of Novel Tyrosinase Inhibitors as Anti-Melanogenic Agents: Design, Synthesis, Structural Insights and Biological Profile. Eur. J. Med. Chem..

[36] Maurus R., Begum A., Williams L.K., Fredriksen J.R., Zhang R., Withers S.G., Brayer G.D. (2008). Alternative Catalytic Anions Differentially Modulate Human α-Amylase Activity and Specificity. Biochemistry.

[37] Karade S.S., Hill M.L., Kiappes J.L., Manne R., Aakula B., Zitzmann N., Warfield K.L., Treston A.M., Mariuzza R.A. (2021). N-Substituted Valiolamine Derivatives as Potent Inhibitors of Endoplasmic Reticulum α-Glucosidases I and II with Antiviral Activity. J. Med. Chem..

[38] Martínez-Rosell G., Giorgino T., De Fabritiis G. (2017). PlayMolecule ProteinPrepare: A Web Application for Protein Preparation for Molecular Dynamics Simulations. J. Chem. Inf. Model..

[39] Pettersen E.F., Goddard T.D., Huang C.C., Couch G.S., Greenblatt D.M., Meng E.C., Ferrin T.E. (2004). UCSF Chimera—A Visualization System for Exploratory Research and Analysis. J. Comput. Chem..

[40] Morris G.M., Lim-Wilby M., Kukol A. (2008). Molecular Docking. Molecular Modeling of Proteins.

[41] Buran K., Kalay Ş., Doğanay D., Öner N., Uba A.I., Zengin G. (2026). Design, Synthesis and Biological Activities of Gold-Conjugated Piperazine-Dithiocarbamates: Antimicrobial and Tyrosinase Inhibitory Activities. J. Mol. Struct..

[42] Ramírez D., Caballero J. (2018). Is It Reliable to Take the Molecular Docking Top Scoring Position as the Best Solution without Considering Available Structural Data?. Molecules.

[43] Uba A.I., Weako J., Keskin Ö., Gürsoy A., Yelekçi K. (2020). Examining the Stability of Binding Modes of the Co-Crystallized Inhibitors of Human HDAC8 by Molecular Dynamics Simulation. J. Biomol. Struct. Dyn..

