# Supplementary material for: Synthesis, Biological Evaluation, Molecular Docking and DFT Studies of Electronically Tuned Benzylidene-Based Schiff Bases
Source: Molecules. 2026 Oct 2;31(19):3519. doi: 10.3390/molecules31193519 (PMC13666581; doi:10.3390/molecules31193519)
Supplement: Supplementary file 1 [file molecules-31-03519-s001.zip › molecules-4585747-supplementary.pdf]

SUPPLEMENTARY INFORMATION

Synthesis, Biological Evaluation, Molecular Docking and DFT Studies of Electronically Tuned Benzylidene-Based Schiff Bases

Kerem Buran [1, 2]*, Elmas Rumeysa Yıldırım [1, 3], Nazmiye Öner [4], Derya Doğanay [5], Esra Mertoğlu [6], Abdullahi Ibrahim Uba [7], Gokhan Zengin [8]

[1] Department of Pharmaceutical Chemistry, Hamidiye Faculty of Pharmacy, University of Health Sciences, Istanbul 34668, Turkey

[2] Experimental Medicine Application & Research Center, Validebağ Research Park, University of Health Sciences, Istanbul, Turkey

[3] Istanbul University, Institute of Graduate Studies in Health Sciences, Department of Pharmaceutical Chemistry, Istanbul, Turkey

[4] Opticianry Program, Hamidiye Vocational School of Health Services, University of Health Sciences, Istanbul 34668, Turkey

[5] Department of Pharmaceutical Microbiology, Hamidiye Faculty of Pharmacy, University of Health Sciences, Istanbul 34668, Turkey

[6] Department of Phytotherapy, Hamidiye Institute of Health Sciences, University of Health Science, Istanbul 34668, Turkey

[7] Department of Biostatistics and Medical Informatics, Faculty of Medicine, İstinye University, 34396 Istanbul, Turkey

[8] Department of Biology, Science Faculty, Selcuk University, Konya, 42130, Turkey

Corresponding author: Kerem Buran, kerem.buran@sbu.edu.tr

Compound 1: N-benzylideneaniline, $C_{13}H_{11}N$

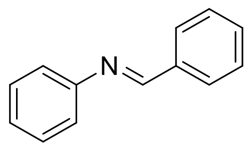

Compound 2: N-(4-methoxybenzylidene)aniline, $C_{14}H_{13}NO$

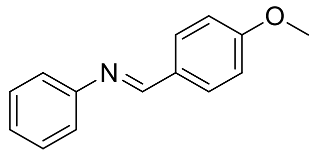

Compound 3: N,N-dimethyl-4-((phenylimino)methyl)aniline, $C_{15}H_{16}N_2$

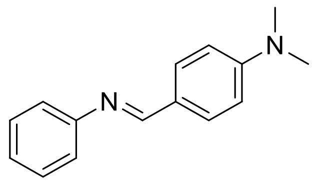

Compound 4: N-benzylidene-4-chloroaniline, $C_{13}H_{10}ClN$

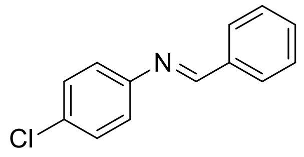

Compound 5: 4-chloro-N-(4-methoxybenzylidene)aniline, $C_{14}H_{12}ClNO$

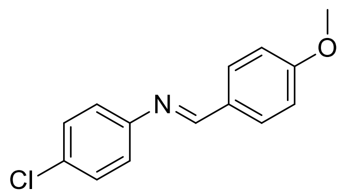

Compound 6: 4-(((4-chlorophenyl)imino)methyl)-N,N-dimethylaniline, $C_{15}H_{15}ClN_2$

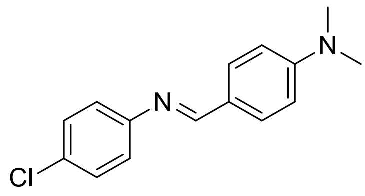

Compound 1

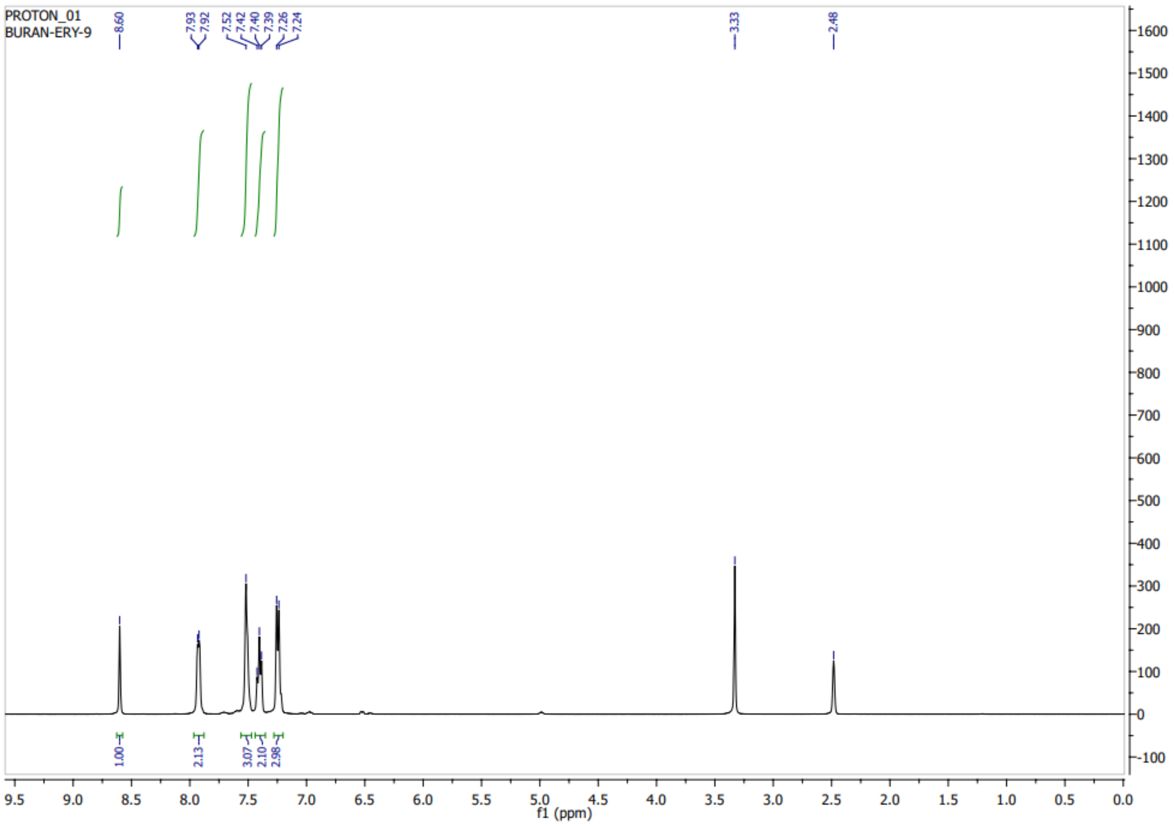
PROTON_01
BURAN-ERY-9
8.60
7.93
7.92
7.52
7.42
7.40
7.39
7.26
7.24
3.33
2.48
1.00
2.13
3.07
2.10
2.98
f1 (ppm)

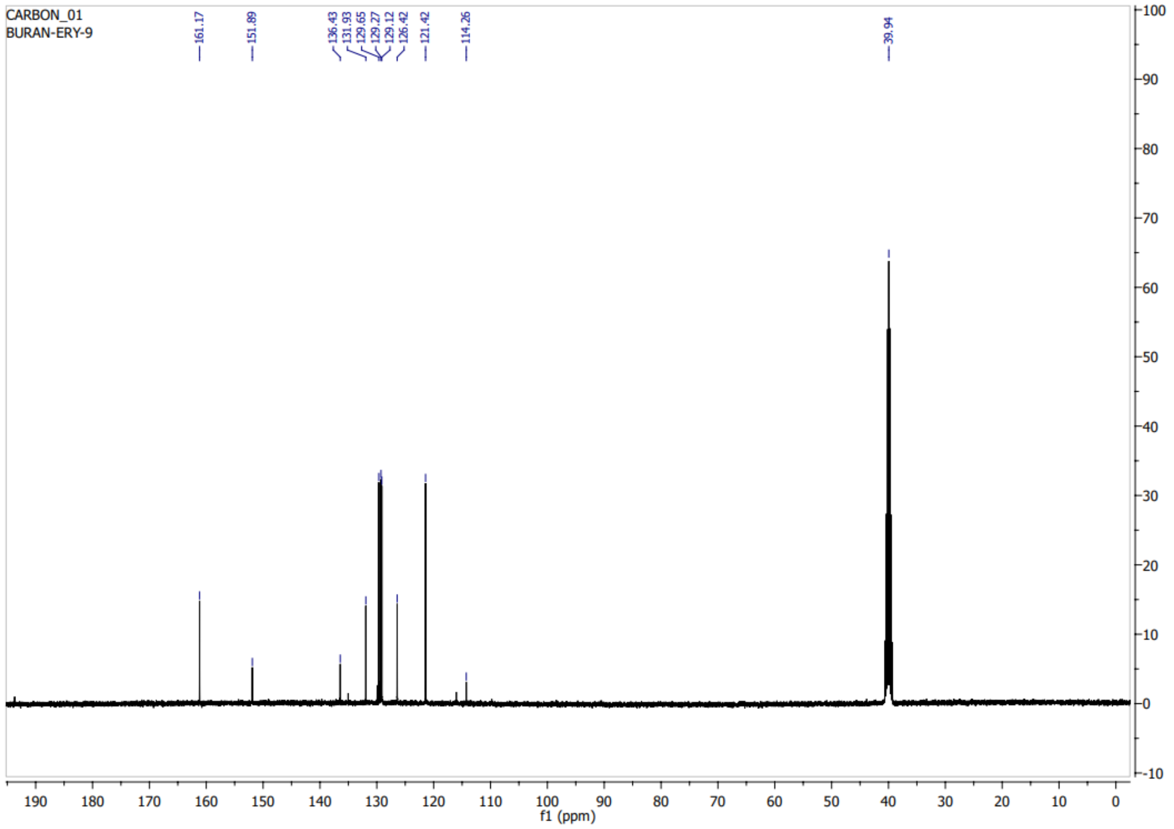
CARBON_01
BURAN-ERY-9
161.17
151.89
136.43
131.93
129.65
129.27
129.12
126.42
121.42
114.26
39.94
f1 (ppm)

Compound 2

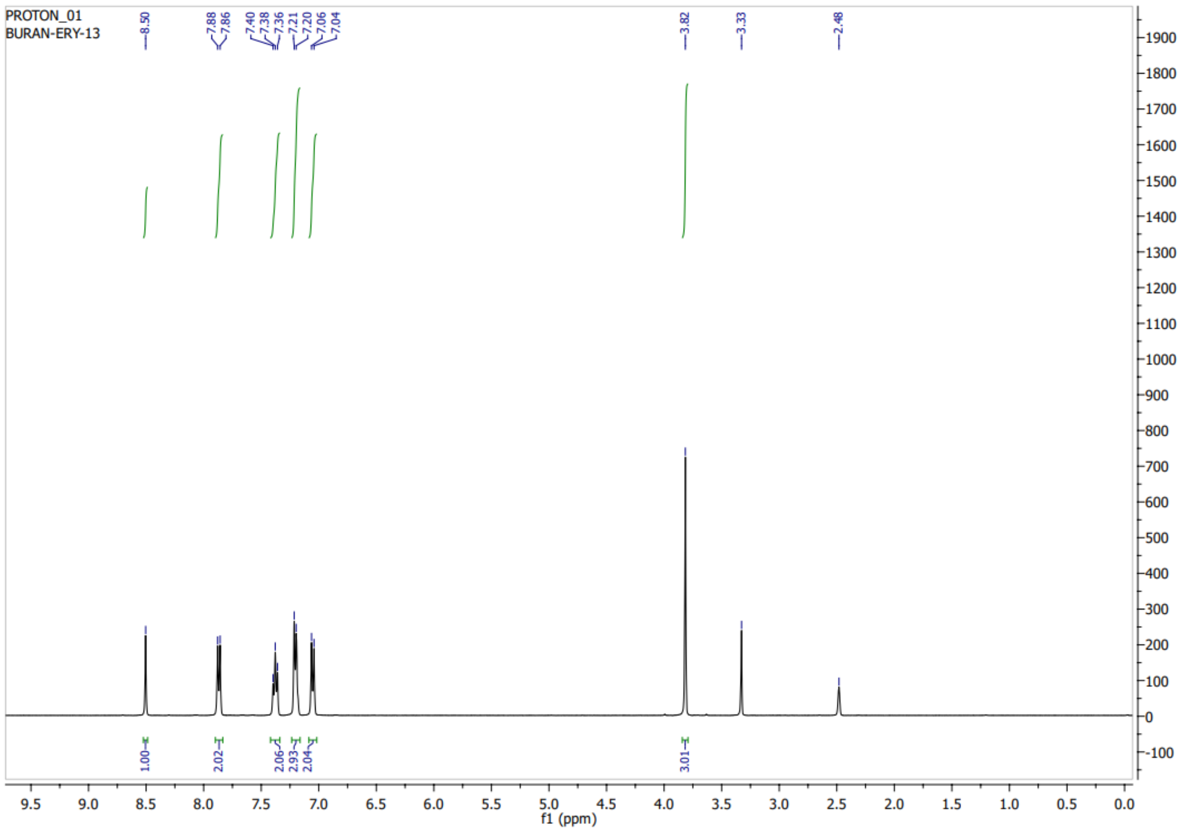
PROTON_01
BURAN-ERY-13
8.50
7.88
7.86
7.40
7.38
7.36
7.21
7.20
7.06
7.04
3.82
3.33
2.48
1.00
2.02
2.06
2.93
2.04
3.01
f1 (ppm)

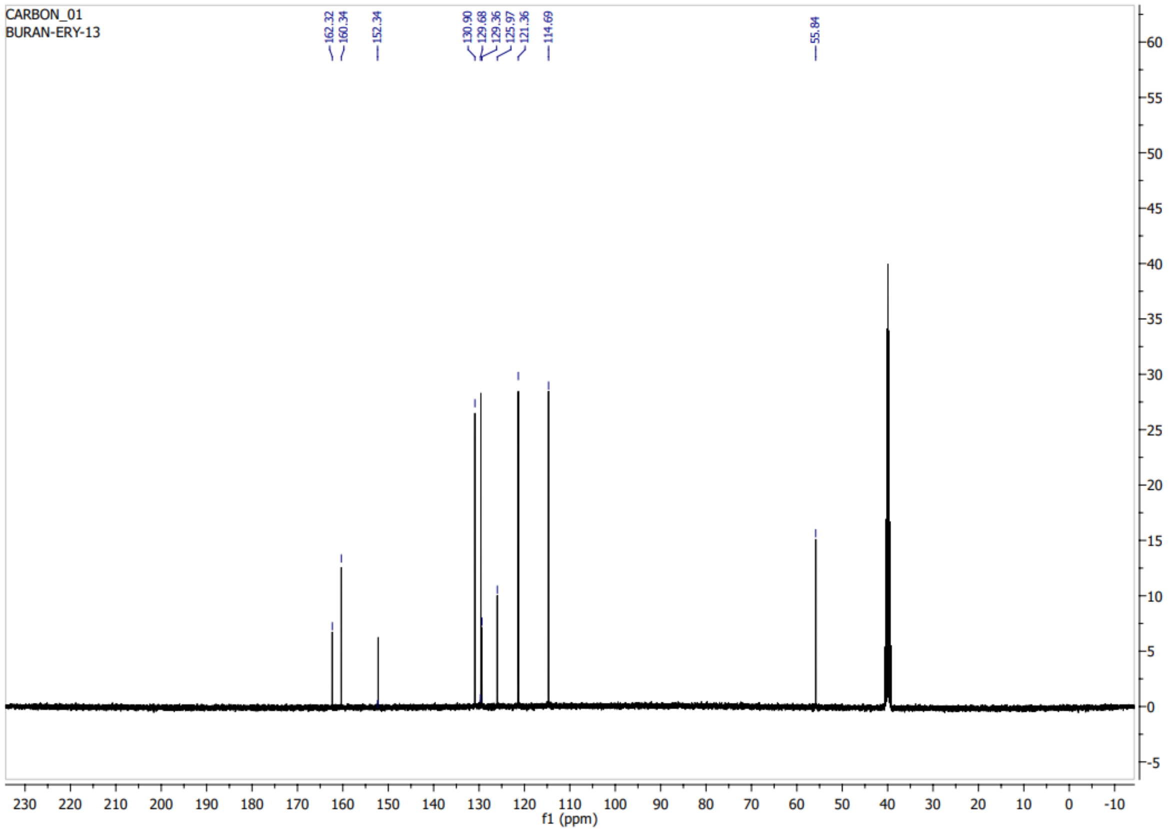
CARBON_01
BURAN-ERY-13
162.32
160.34
152.34
130.90
129.68
129.36
125.97
121.36
114.69
55.84
f1 (ppm)

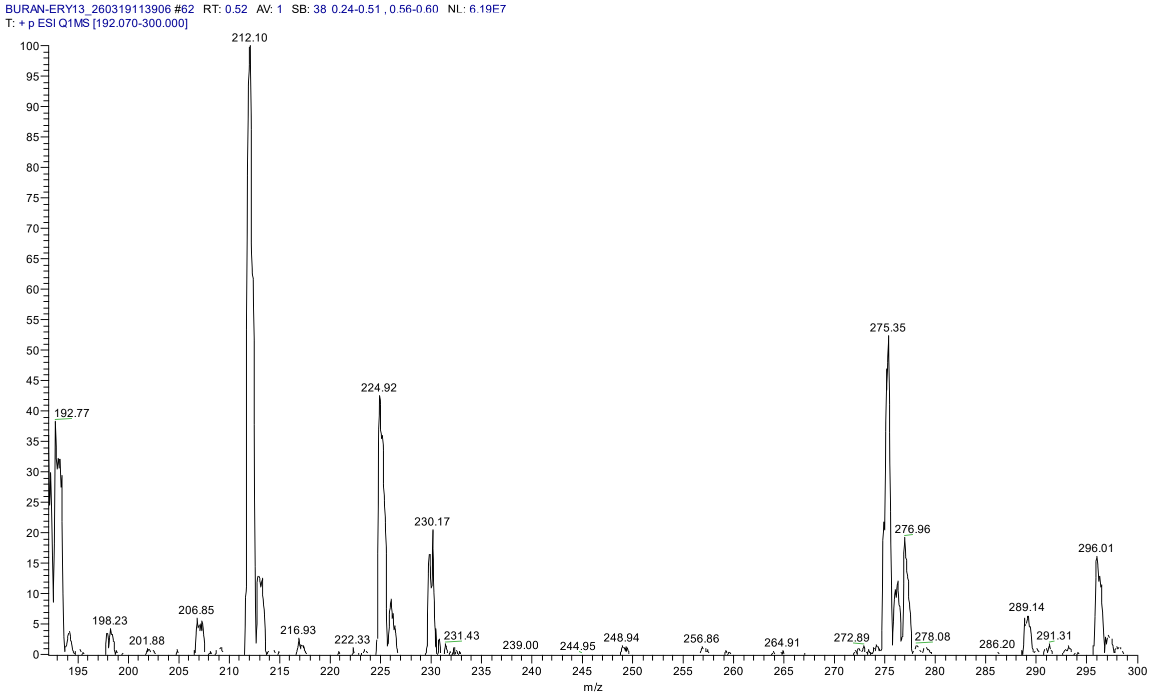

BURAN-ERY13_260319113906 #62 RT: 0.52 AV: 1 SB: 38 0.24-0.51 , 0.56-0.60 NL: 6.19E7
T: + p ESI Q1MS [192.070-300.000]
212.10
275.35
224.92
192.77
230.17
276.96
296.01
289.14
198.23
206.85
216.93
m/z

Compound 3

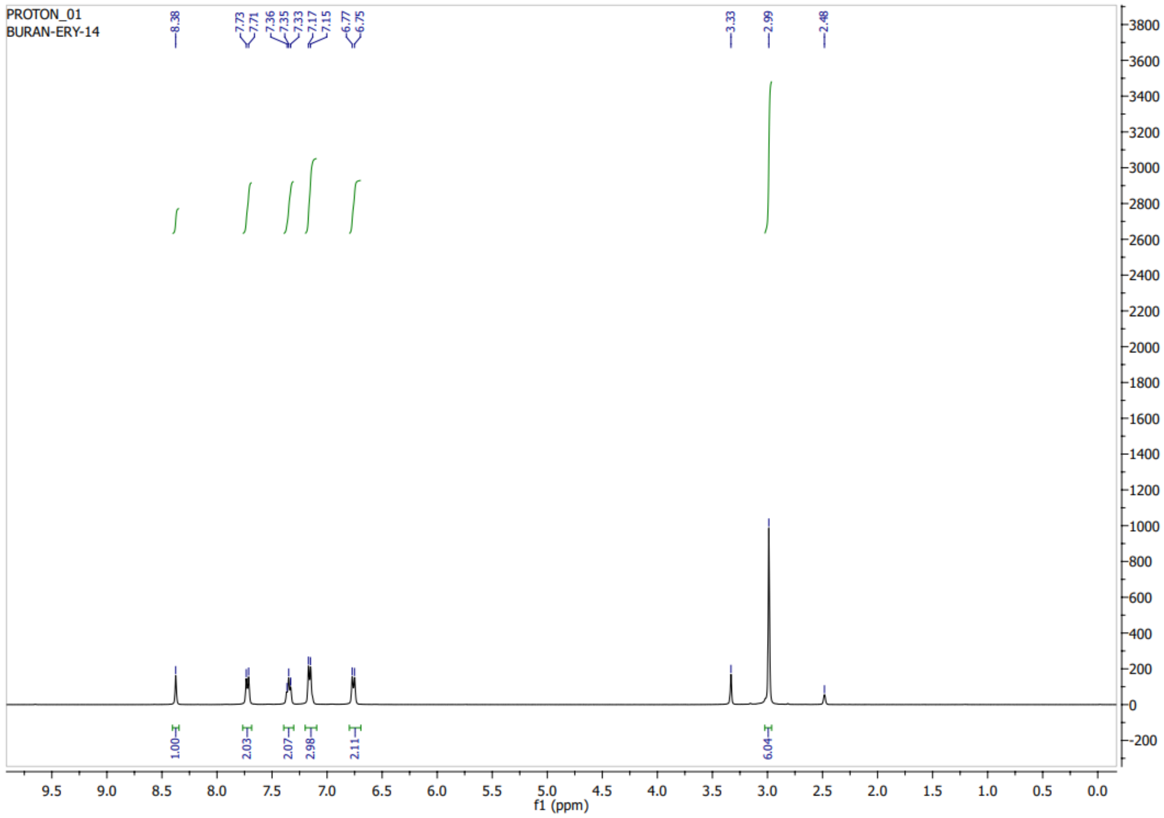

PROTON_01
BURAN-ERY-14
8.38
7.73
7.71
7.36
7.35
7.33
7.17
7.15
6.77
6.75
3.33
2.99
2.48
1.00
2.03
2.07
2.98
2.11
6.04
f1 (ppm)

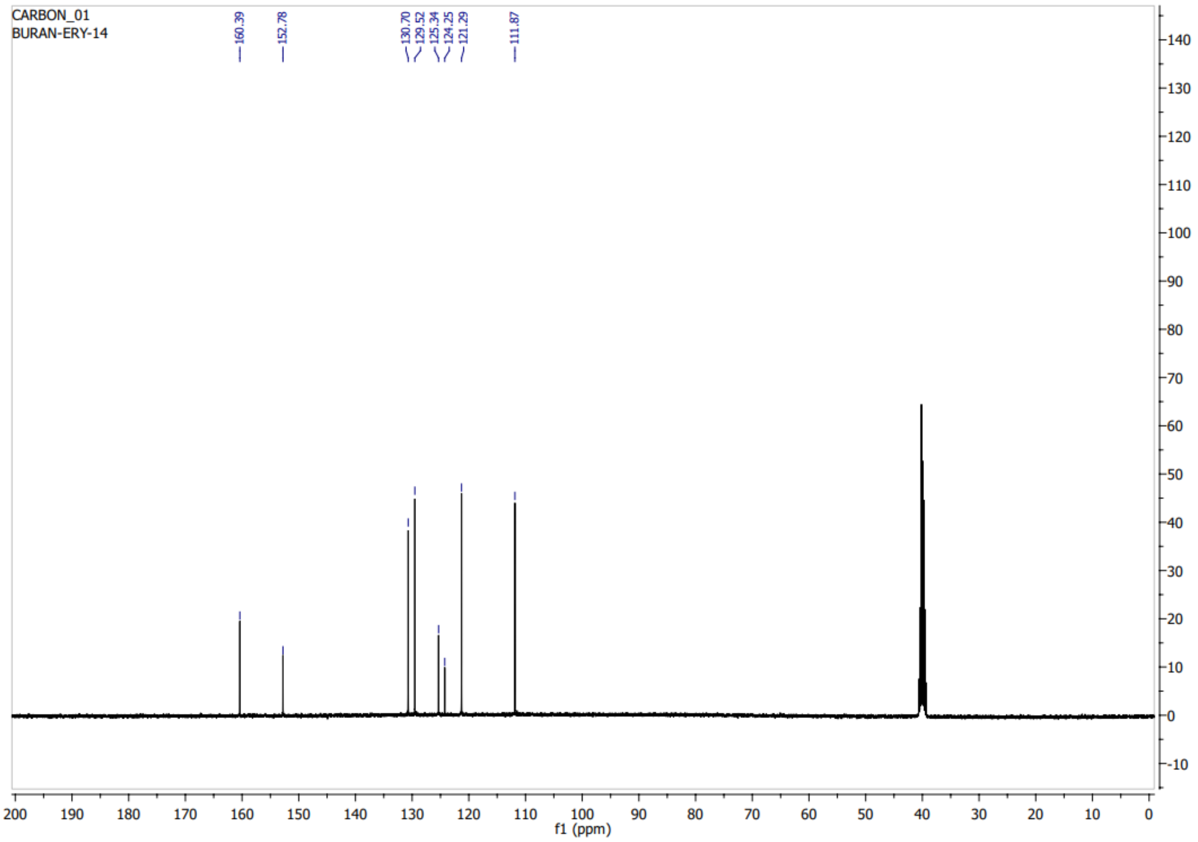
CARBON_01
BURAN-ERY-14
160.39
152.78
130.70
129.52
125.34
124.25
121.29
111.87
f1 (ppm)

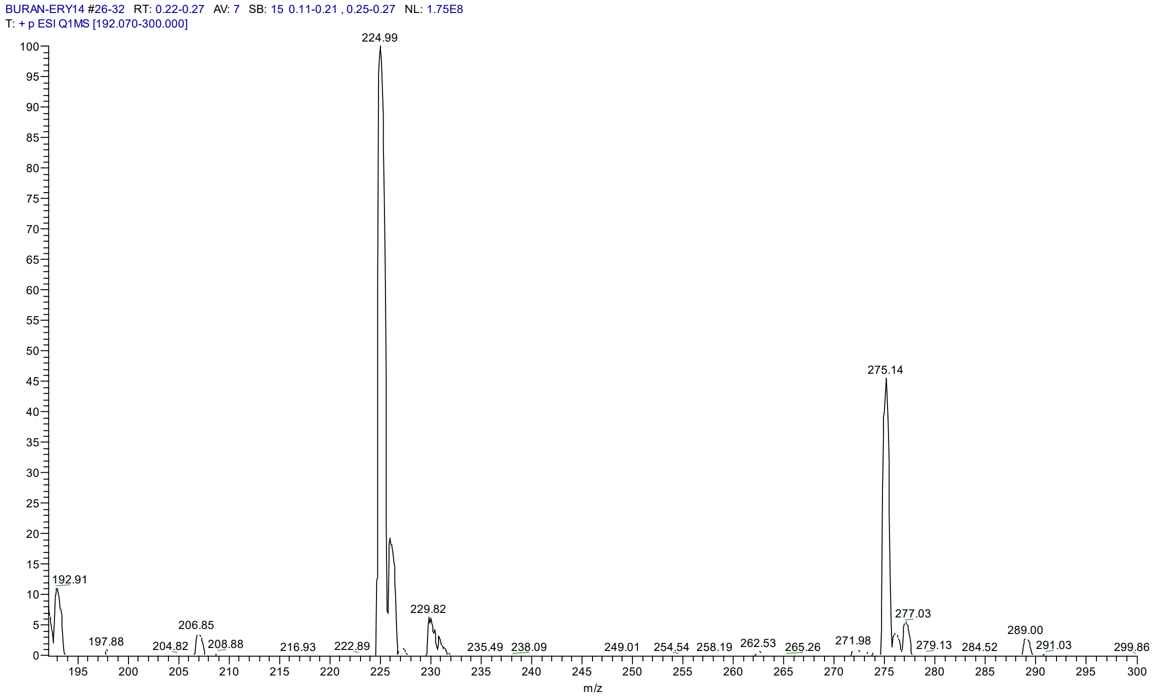
BURAN-ERY14 #26-32 RT: 0.22-0.27 AV: 7 SB: 15 0.11-0.21 , 0.25-0.27 NL: 1.75E8
T: + p ESI Q1MS [192.070-300.000]
224.99
275.14
192.91
229.82
206.85
277.03
289.00
m/z

Compound 4

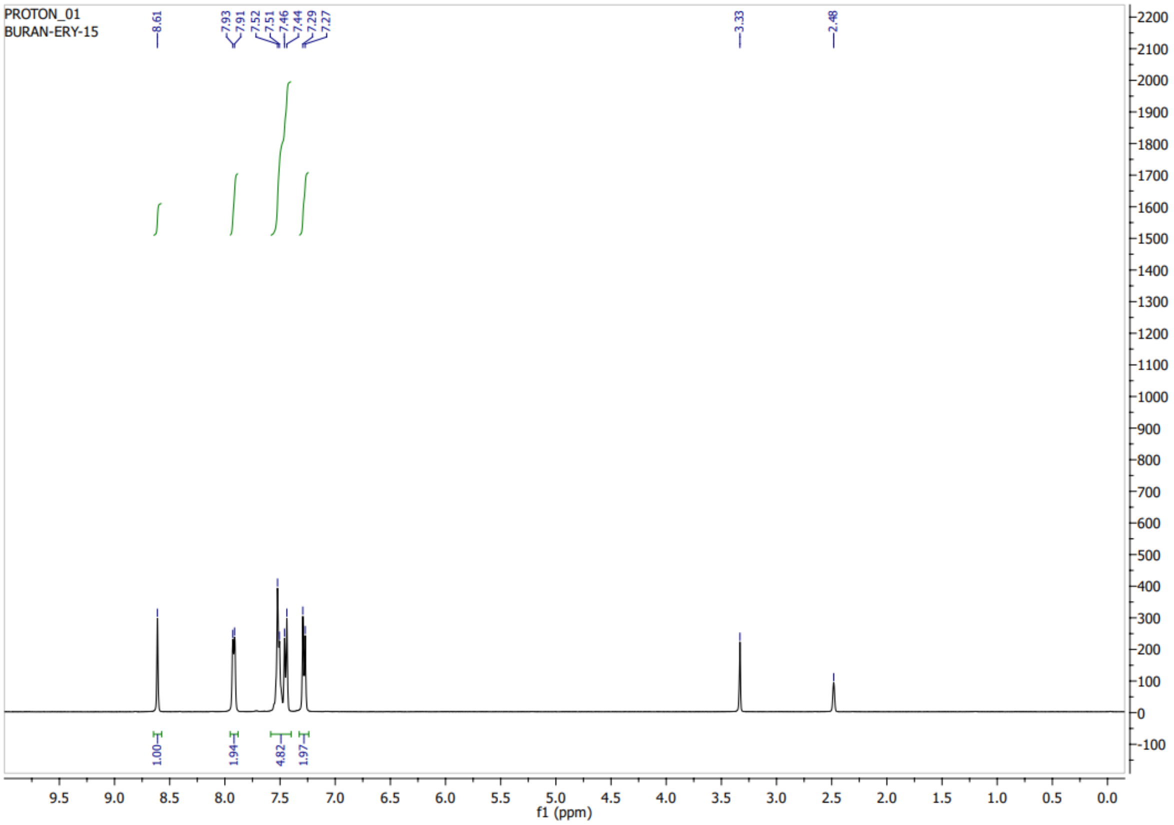

PROTON_01
BURAN-ERY-15
8.61
7.93
7.91
7.52
7.51
7.46
7.44
7.29
7.27
3.33
2.48
1.00
1.94
4.82
1.97
f1 (ppm)

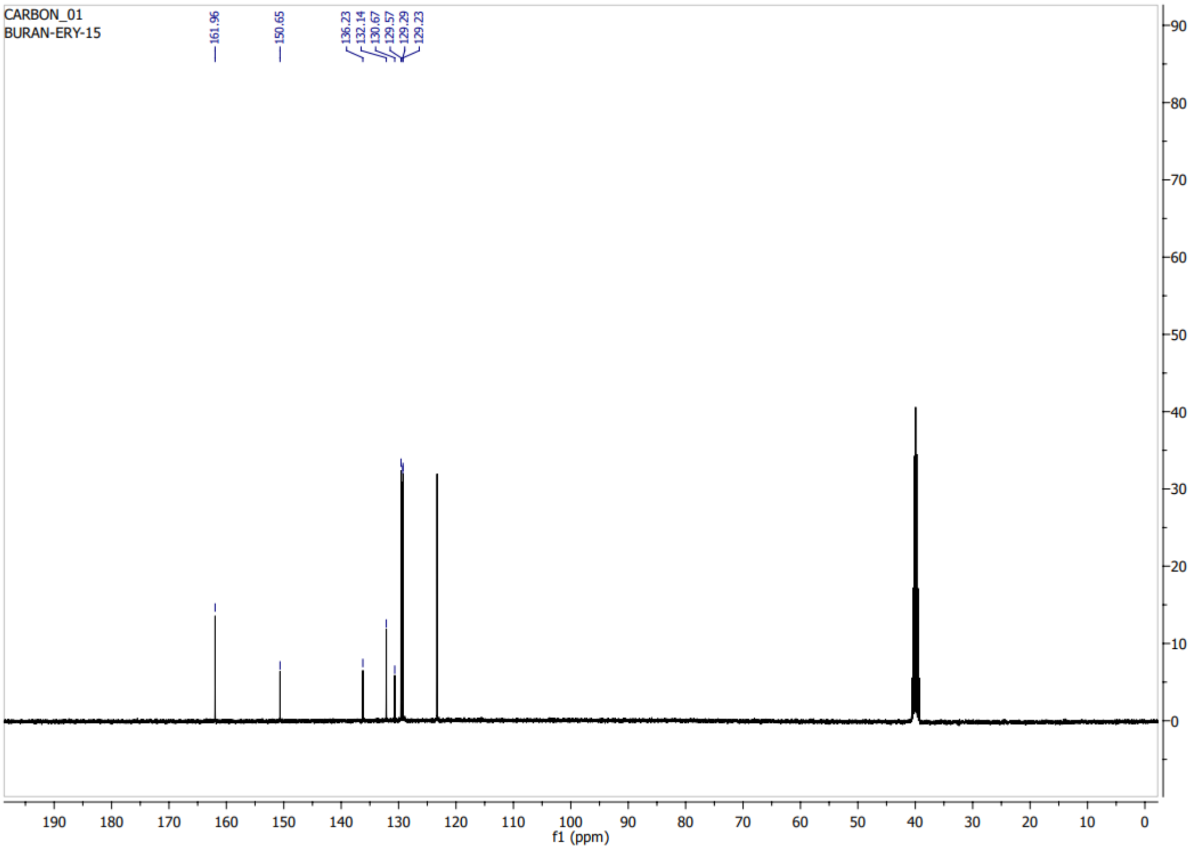

CARBON_01
BURAN-ERY-15
161.96
150.65
136.23
132.14
130.67
129.57
129.29
129.23
f1 (ppm)

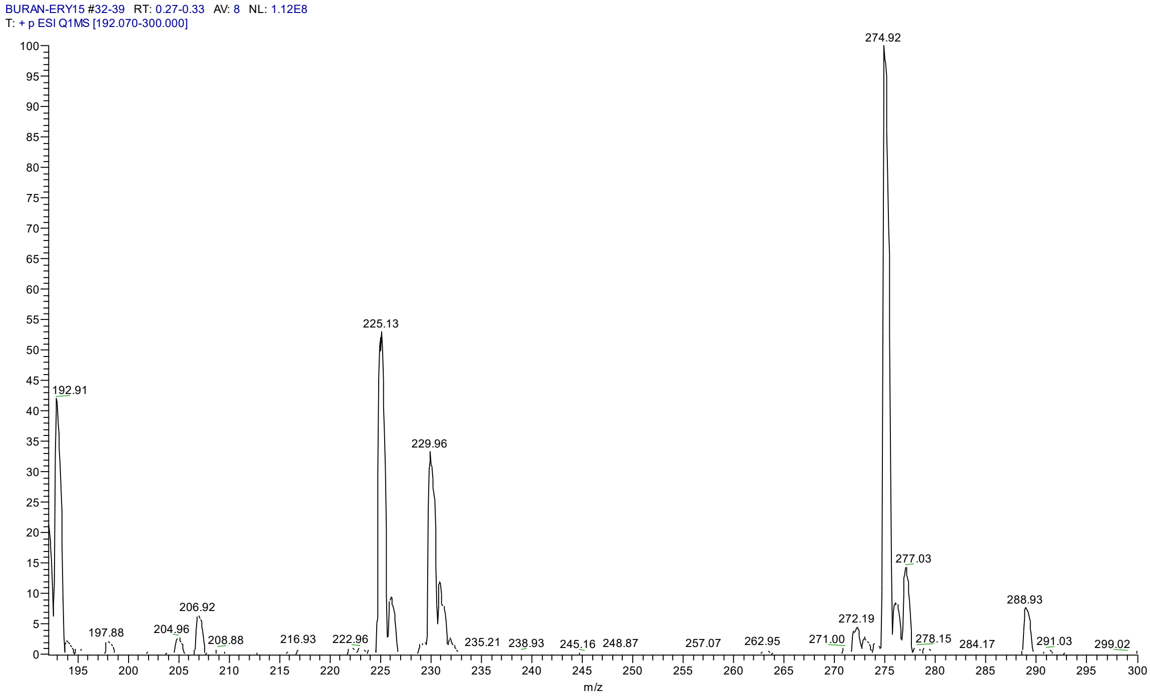
BURAN-ERY15 #32-39 RT: 0.27-0.33 AV: 8 NL: 1.12E8
T: + p ESI Q1MS [192.070-300.000]
274.92
225.13
229.96
192.91
277.03
288.93
206.92
272.19
m/z

Compound 5

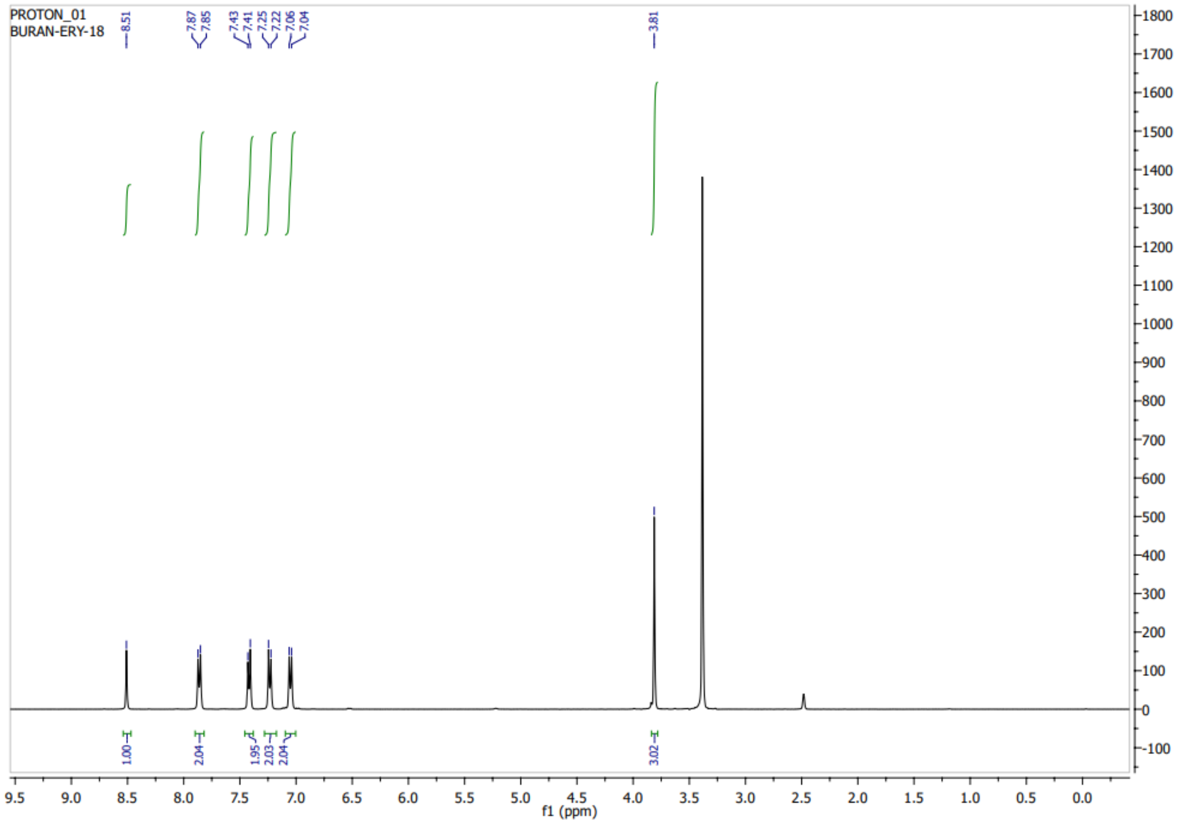
PROTON_01
BURAN-ERY-18
8.51
7.87
7.85
7.43
7.41
7.25
7.22
7.06
7.04
3.81
1.00
2.04
1.95
2.03
2.04
3.02
f1 (ppm)

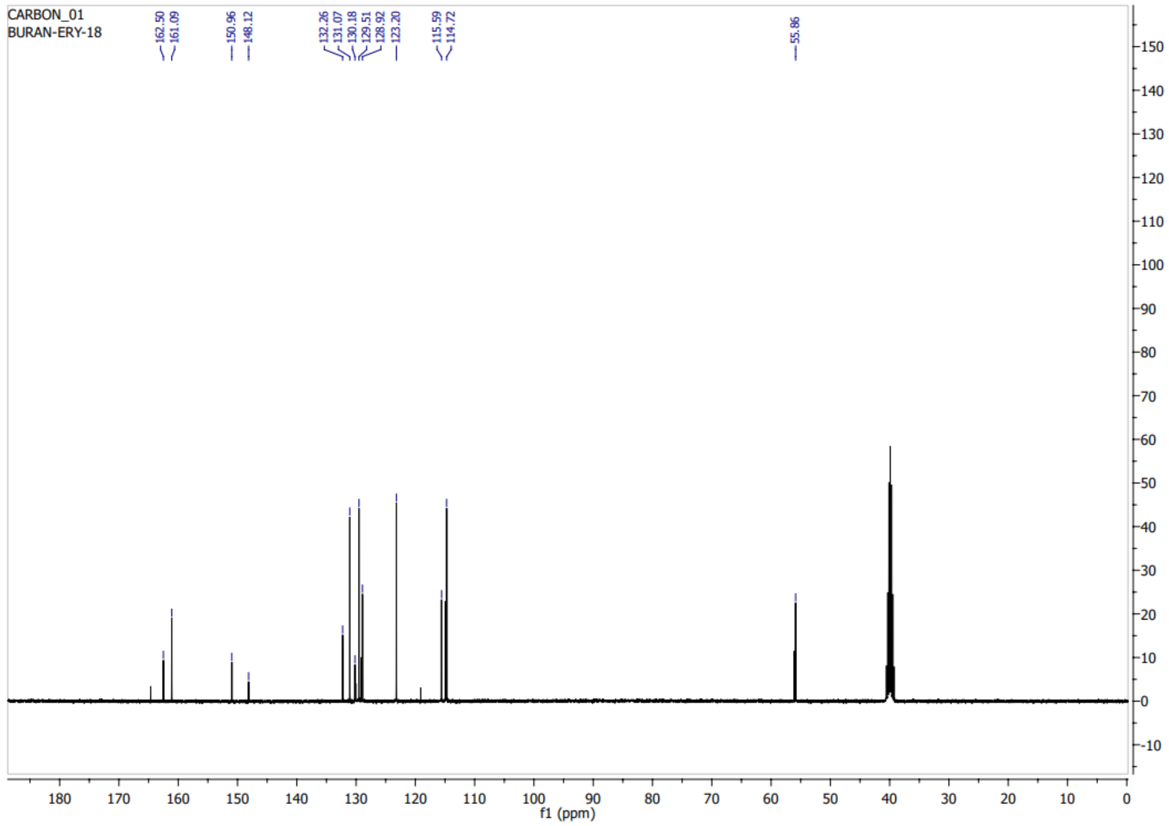
CARBON_01
BURAN-ERY-18
162.50
161.09
150.96
148.12
132.26
131.07
130.18
129.51
128.92
123.20
115.59
114.72
55.86
f1 (ppm)

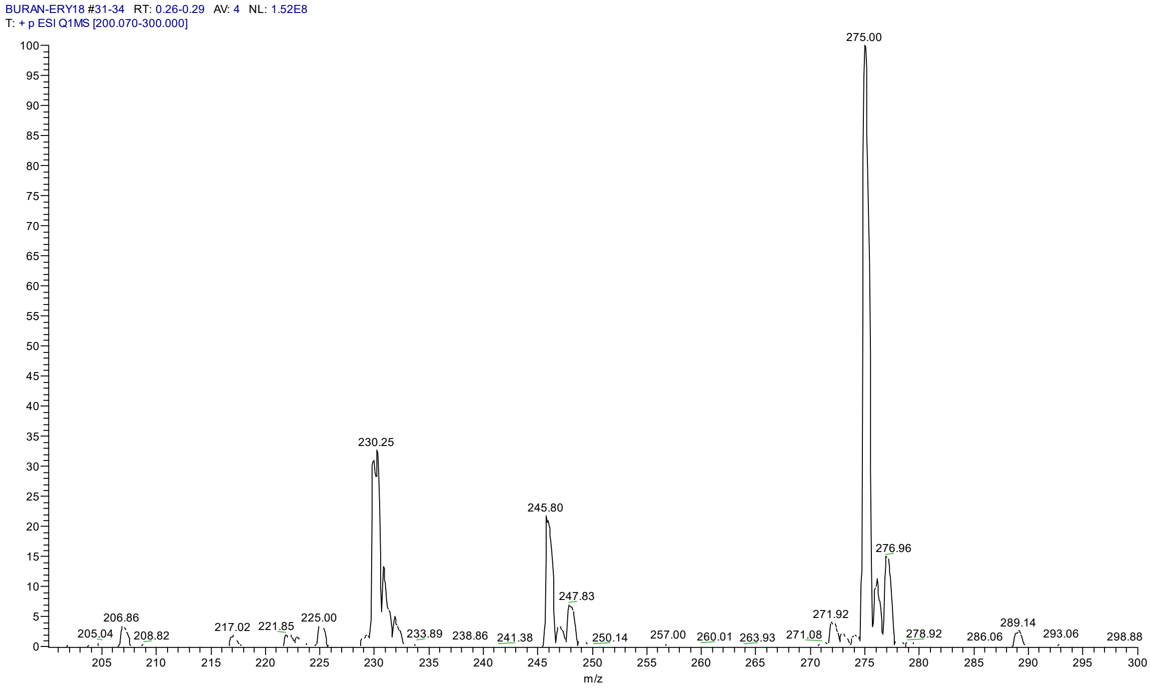
BURAN-ERY18 #31-34 RT: 0.26-0.29 AV: 4 NL: 1.52E8
T: + p ESI Q1MS [200.070-300.000]
275.00
230.25
245.80
276.96
247.83
271.92
289.14
m/z

Compound 6

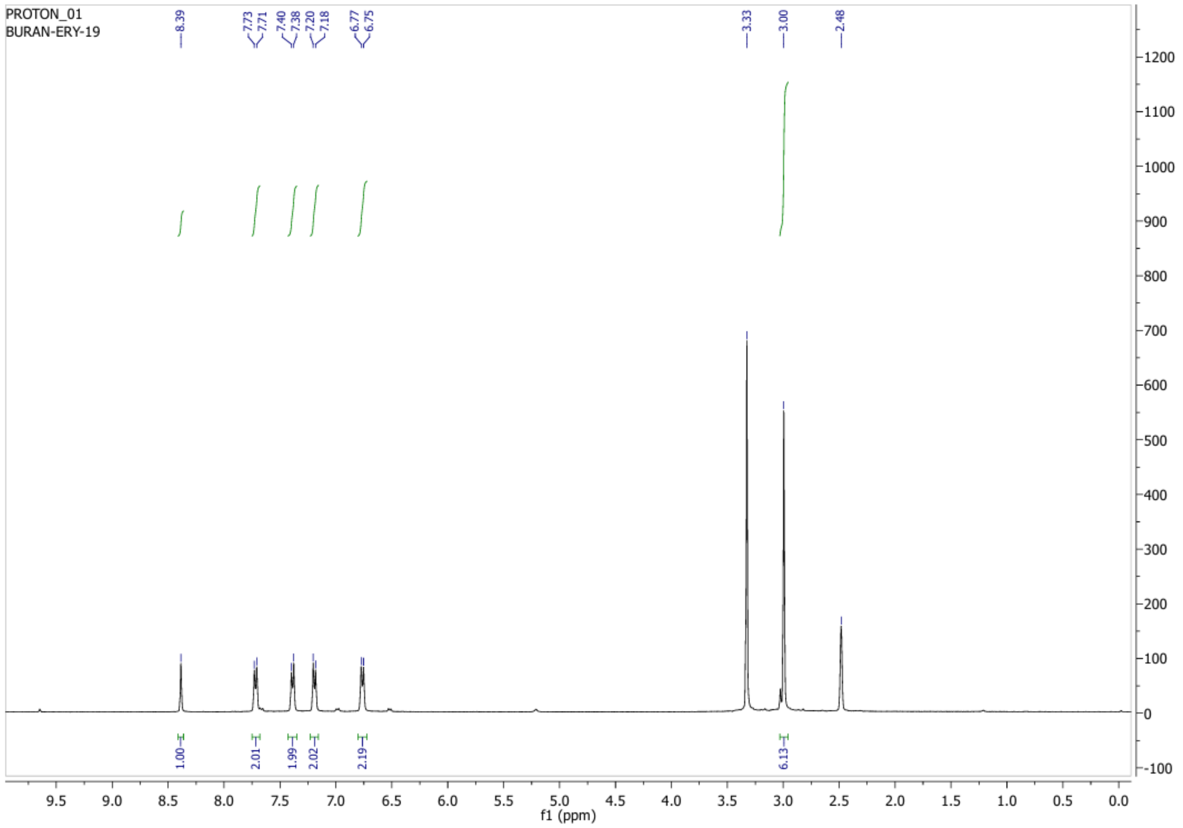
PROTON_01
BURAN-ERY-19
8.39
7.73
7.71
7.40
7.38
7.20
7.18
6.77
6.75
3.33
3.00
2.48
1.00
2.01
1.99
2.02
2.19
6.13
f1 (ppm)

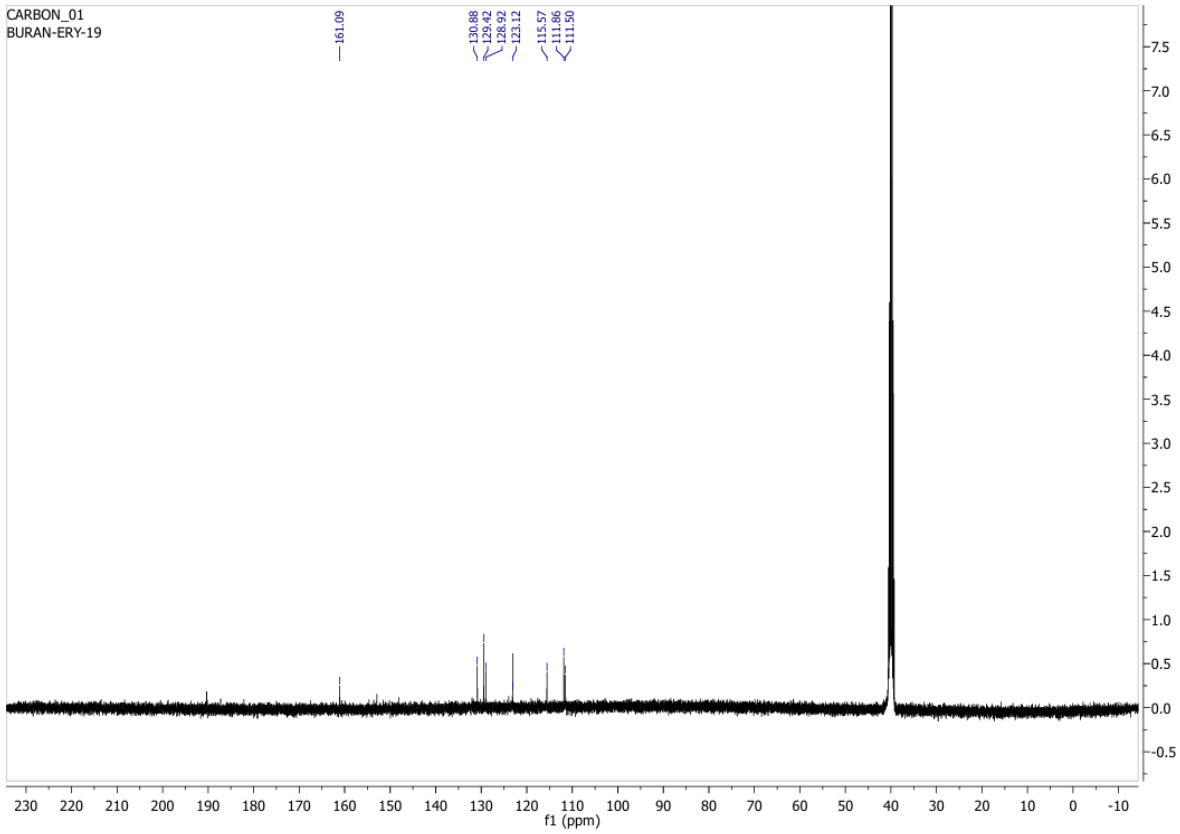
CARBON_01
BURAN-ERY-19
161.09
130.88
129.42
128.92
123.12
115.57
111.86
111.50
f1 (ppm)

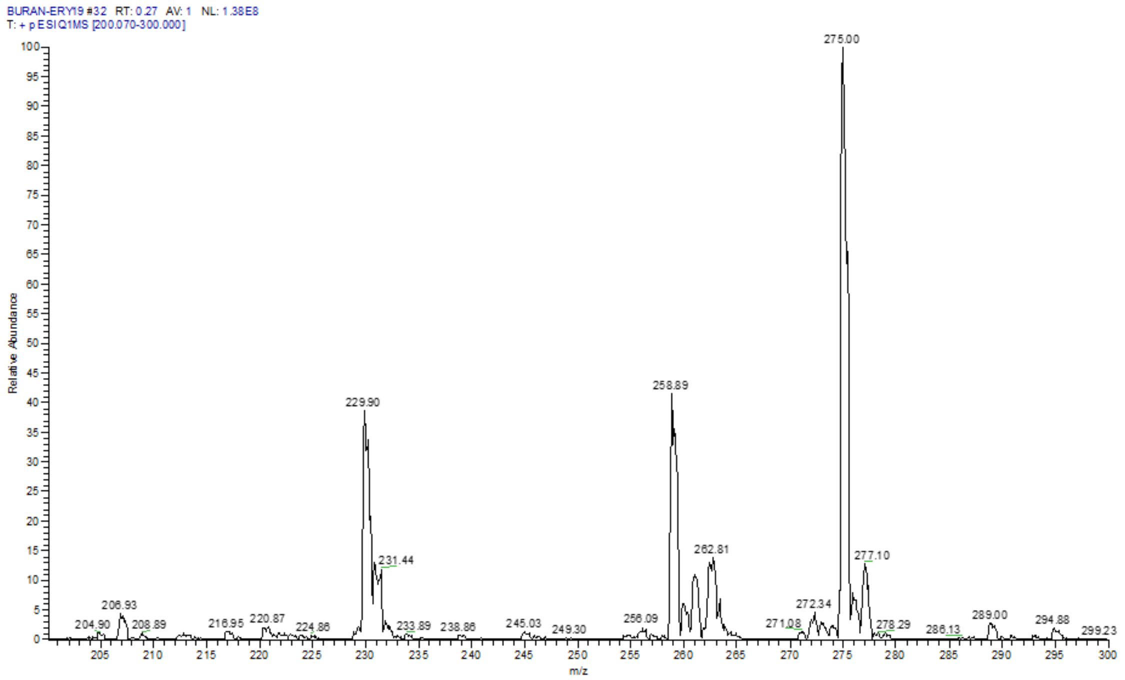

Table S1. The HOMO and LUMO energies, the energy gap (ΔE), the global reactivity descriptors (χ, η, S, I, A, μ, ω) for **1-6** [25].

Parameters	1	2	3	4	5	6
E_{HOMO} (eV)	-6.26	-5.97	-5.41	-6.33	-6.05	-5.51
E_{LUMO} (eV)	-2.08	-1.74	-1.46	-2.26	-1.91	-1.64
ΔE (eV)	4.18	4.24	3.94	4.07	4.14	3.87
I (eV)	6.26	5.97	5.41	6.33	6.05	5.51
A (eV)	2.08	1.74	1.46	2.26	1.91	1.64
χ (eV)	4.17	3.85	3.44	4.29	3.98	3.58
μ (eV)	-4.17	-3.85	-3.44	-4.29	-3.98	-3.58
η (eV)	2.09	2.12	1.97	2.04	2.07	1.93
S (1/eV)	0.24	0.24	0.25	0.25	0.24	0.26
ω (eV)	4.16	3.51	2.99	4.53	3.83	3.30

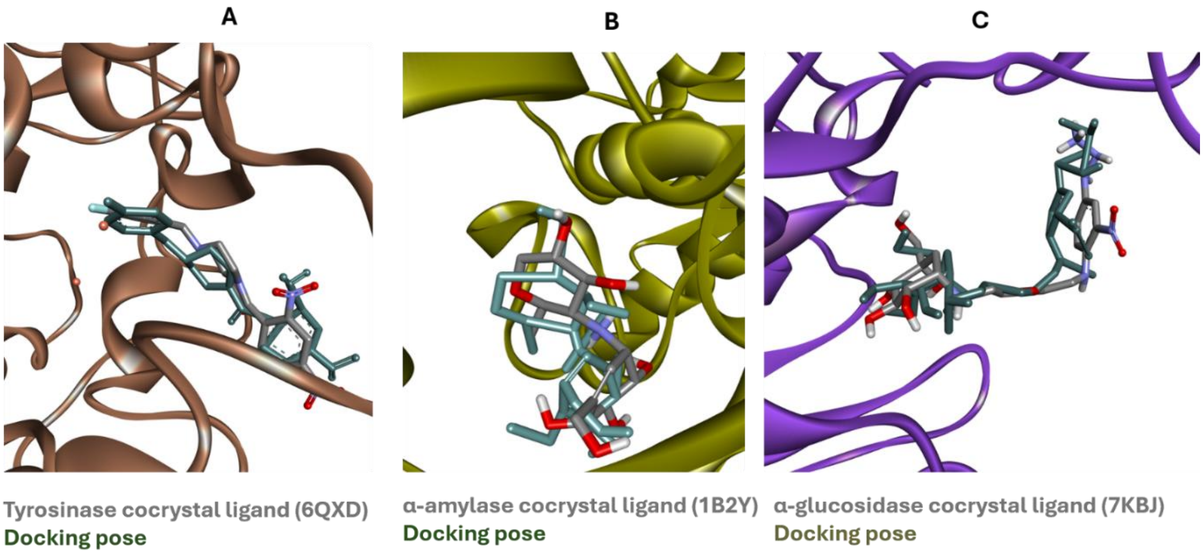

Figure S1. Comparison of co-crystallized ligand poses and corresponding docking poses for tyrosinase (6QXD), α-amylase (1B2Y), and α-glucosidase (7KBI)
